# Prostaglandin E2 Antagonizes TGF-β Actions During the Differentiation of Monocytes Into Dendritic Cells

**DOI:** 10.3389/fimmu.2018.01441

**Published:** 2018-06-22

**Authors:** Federico Remes Lenicov, Ana Luz Paletta, Melina Gonzalez Prinz, Augusto Varese, Clara E. Pavillet, Álvaro Lopez Malizia, Juan Sabatté, Jorge Raul Geffner, Ana Ceballos

**Affiliations:** Instituto de Investigaciones Biomédicas en Retrovirus y SIDA (INBIRS), Universidad de Buenos Aires, Buenos Aires, Argentina

**Keywords:** prostaglandin E2, monocytes, dendritic cells, TGF-β, inflammation, IL-10

## Abstract

Inflammatory dendritic cells (DCs) are a distinct subset of DCs that derive from circulating monocytes infiltrating injured tissues. Monocytes can differentiate into DCs with different functional signatures, depending on the presence of environment stimuli. Among these stimuli, transforming growth factor-beta (TGF-β) and prostaglandin E2 (PGE2) have been shown to modulate the differentiation of monocytes into DCs with different phenotypes and functional profiles. In fact, both mediators lead to contrasting outcomes regarding the production of inflammatory and anti-inflammatory cytokines. Previously, we have shown that human semen, which contains high concentrations of PGE2, promoted the differentiation of DCs into a tolerogenic profile through a mechanism dependent on signaling by E-prostanoid receptors 2 and 4. Notably, this effect was induced despite the huge concentration of TGF-β present in semen, suggesting that PGE2 overrides the influence exerted by TGF-β. No previous studies have analyzed the joint actions induced by PGE2 and TGF-β on the function of monocytes or DCs. Here, we analyzed the phenotype and functional profile of monocyte-derived DCs differentiated in the presence of TGF-β and PGE2. DC differentiation guided by TGF-β alone enhanced the expression of CD1a and abrogated LPS-induced expression of IL-10, while differentiation in the presence of PGE2 impaired CD1a expression, preserved CD14 expression, abrogated IL-12 and IL-23 production, stimulated IL-10 production, and promoted the expansion of FoxP3+ regulatory T cells in a mixed lymphocyte reaction. Interestingly, DCs differentiated in the presence of TGF-β and PGE2 showed a phenotype and functional profile closely resembling those induced by PGE2 alone. Finally, we found that PGE2 inhibited TGF-β signaling through an action exerted by EP2 and EP4 receptors coupled to cyclic AMP increase and protein kinase A activity. These results indicate that PGE2 suppresses the influence exerted by TGF-β during DC differentiation, imprinting a tolerogenic signature. High concentrations of TGF-β and PGE2 are usually found in infectious, autoimmune, and neoplastic diseases. Our observations suggest that in these scenarios PGE2 might play a mandatory role in the acquisition of a regulatory profile by DCs.

## Introduction

Dendritic cells (DCs) comprise a heterogeneous group of cells characterized by their ability not only to induce T cell activation but also to silence T cell immune responses. So called “inflammatory” DCs are a distinct subset of DCs derived from monocytes that infiltrate pathogen-induced or sterile inflammation sites (1). Inflammatory DCs show high plasticity, which enables them to induce the differentiation of CD4+ T cells into different effector profiles (2–5). They can also differentiate into tolerogenic DCs to keep the immune response under control (6). Microenvironmental signals sensed by infiltrating monocytes during their differentiation determine the functional profile of DCs (7), although it is still unclear how the acquisition of a particular signature is regulated *in vivo*.

Transforming growth factor-β (TGF-β) is found at increased levels at inflammatory sites, either during acute or chronic stages of inflammation, as well as in tumor microenvironments. TGF-β is usually associated with the resolution of inflammatory process by dampening the activation of T lymphocytes through different mechanisms (8). For instance, TGF-β is known to suppress the function of effector CD4+ and CD8+ T cells, to promote the activation of naïve CD4+ T cells into a regulatory profile and to suppress the activation of natural killer cells (9–11). The pleiotropic nature of TGF-β action is evident when analyzing its effect on myeloid cells. Loss of TGF-β signaling specifically in conventional DCs promotes autoimmune encephalomyelitis and spontaneous multiorgan autoimmunity (12, 13), without affecting DC maturation and IL-12 secretion in the scenario of *Leishmania* infection (13, 14). In the early periods of inflammation, TGF-β appears to act as a pro-inflammatory agent by recruiting and activating resting monocytes, notably increasing their susceptibility to be activated through the receptor for the Fc portion of IgG type III (CD16) (15, 16). Furthermore, monocytes cultured with TGF-β (together with GM-CSF and IL-4), differentiate toward Langerhans cell (LC)-like DCs that are unable to produce IL-10 but have a great capacity to secrete IL-12 upon stimulation (17, 18).

Prostaglandin E2 (PGE2), a pleotropic molecule derived from arachidonic acid metabolism, is another ubiquitous immunomodulator known to influence differentiation of DCs. Its concentration rapidly increases in acute inflammatory processes (19, 20), promoting local vasodilation, increasing microvascular permeability, and favoring the extravasation of blood granulocytes and the activation of mast cells (21). Paradoxically, PGE2 is also known for a wide range of immunosuppressive functions like inhibition of neutrophil and NK cell activation, inhibition of T cell proliferation, and abolishing secretion of inflammatory factors like TNF-α and IL-12 from monocytes, macrophages, and DCs (22–24). In the course of DC differentiation induced by GM-CSF and IL-4, PGE2 has been shown to promote the development of myeloid-derived suppressor cells (MDSCs) (25). Interestingly, contrasting with the actions mediated by TGF-β, PGE2 has been shown to enhance IL-10 expression and abrogate IL-12 production by DCs (21).

Because high concentrations of TGF-β and PGE2 are found in the course of infectious, autoimmune, and neoplastic diseases, previous studies have focused on the biological actions induced by the presence of both mediators. It has been reported that PGE2 antagonizes TGF-β signaling in a variety of cell types, including liver stellate cells, mammary epithelial cells, and lung fibroblasts (26–28). For instance, PGE2 is a known anti-fibrotic modulator which counteracts TGF-β pro-fibrotic effects in the liver by acting on stellate cells and fibroblasts (29). Moreover, PGE2 has been shown to inhibit TGF-β-induced mesenchymal epithelial transition (30, 31). On the other hand, it has also been reported that PGE2 can act synergically with TGF-β. In this regard, TGF-β has shown to synergize with PGE2 in the induction of FoxP3 expression in CD4+ T lymphocytes as well as in the inhibition of IFN-α secretion by plasmacytoid DCs (32–34). In a previous study, we found that semen, which contains very high levels of TGF-β and PGE2 (35, 36), guided the differentiation of monocytes into tolerogenic DCs through a mechanism dependent on signaling by E-prostanoid receptors 2 and 4 (37). However, there are no previous studies specifically aimed at analyzing the effect of TGF-β and PGE2 acting together on monocytes or DCs.

In this work, we found that the presence of PGE2 at the onset of the differentiation of monocytes into DCs antagonizes phenotypic and functional signatures induced by TGF-β. We observed that PGE2, in a concentration-dependent manner, skewed the CD1a+ CD14− phenotype stimulated by TGF-β toward a CD1a− CD14+ phenotype. More importantly, we found that the presence of PGE2, in concentrations as low as 10^−7^ M, defined the functional properties of DCs, antagonizing the influence of TGF-β. In fact, PGE2 reversed TGF-β-mediated inhibition of IL-10 and abrogated IL-12 and IL-23 production, leading to expansion of FoxP3+ regulatory T cells in a mixed lymphocyte reaction. We also found that PGE2 interfered with TGF-β signaling by increasing cyclic adenosine mono-phosphate (cAMP), without affecting Smad2 and Smad3 entry into the nucleus, in agreement with a mechanism that was previously described in fibroblasts (38). Our results contribute to the understanding of monocyte fate in inflammatory and tumoral contexts, and suggest that the presence of PGE2 could mask the pro-inflammatory actions exerted by TGF-β on monocytes, favoring the acquisition of a tolerogenic signature by monocyte-derived DCs.

## Materials and Methods

### Reagents

LPS from *Escherichia coli* O111:B4, PHA from *Phaseolus vulgaris*, H-89 [protein kinase A (PKA) inhibitor], forskolin (adenylate cyclase activator), and PGE2 were obtained from Sigma-Aldrich. AS-605240 [phosphatidylinositol-3-phosphate kinase (PI3K) inhibitor], PF-04418948 (EP2 receptor antagonist), and L-161,962 (EP4 receptor antagonist) were purchased from Cayman Chemical. Ficoll-Hypaque was obtained from GE Healthcare. Recombinant human TGF-β1, recombinant human M-CSF, recombinant human IL-4, and recombinant human GM-CSF were obtained from Miltenyi Biotech.

### Preparation of DCs, Macrophages, and CD4+ T Cells

Buffy coats were obtained from healthy blood donors. Peripheral blood mononuclear cells (PBMCs) were isolated by standard density gradient centrifugation on Ficoll-Hypaque. Monocytes were obtained using CD14 microbeads (Miltenyi Biotec). The purity was checked by flow cytometry analysis using an anti-CD14 mAb and was found to be >95%. To obtain DCs, purified monocytes were cultured for 5 days in RPMI-1640 (Thermo Fisher) supplemented with 10% heat-inactivated fetal calf serum, 50 U/ml penicillin, 50 µg/ml streptomycin, and 0.1 mM nonessential amino acids (complete culture medium; all from Thermo Fisher) at 1 × 10^6^ cells/ml with 10 ng/ml IL-4 and 10 ng/ml GM-CSF, as previously described (39). These cells were labeled as control DCs. For DCs obtained in the presence of TGF-β and/or PGE2, concentrations are indicated in the text. In the figure legends, *n* indicates the number of individual donors of DCs.

For experiments involving treatment with EP2 and EP4 receptor antagonists, monocytes were washed after 24 h of culture, then replated with IL-4 and GM-CSF for the remaining 4 days. To induce maturation, DCs were washed at day 5 of culture and treated with LPS (20 ng/ml).

Macrophages were prepared by plating CD14+ monocytes (purified as described below) with M-CSF (50 ng/ml) for 7 days. CD4+ T cells were purified from monocyte-depleted PBMCs using microbeads for CD4 enrichment cocktail (Miltenyi Biotec).

### T-Cell-DC Co-Cultures

#### Dendritic Cells Were Differentiated for 5 Days as Described Below

Maturation of DCs was induced by treatment with LPS (20 ng/ml) for 24 h. In all cases, DCs were washed, counted, and replated for incubation with freshly isolated allogeneic CD4+ T cells (160,000 cells/well) in U-bottomed 96-well plates (Costar). Thus, co-cultures were always free of exogenous IL-4, GM-CSF, PGE2, or TGF-β.

#### Cell Proliferation Assays by CFSE Staining Dilution

Freshly isolated lymphocytes were labeled with 5,6-carboxyfluorescein succinimidyl ester (CFSE) (Invitrogen) at 1 µM concentration, according to the manufacturer’s instructions. CFSE-stained lymphocytes were coincubated with DCs using a DC:lymphocyte ratio of 1:4. After for 4 days, CD4+ T cells were stained with anti-CD3 mAb. Cellular proliferation was assayed by flow cytometry of CFSE staining in the CD3+ cell population.

Cell proliferation assays by Ki-67 intracytoplasmic staining. CD4+ T cells were coincubated with DCs for 2 days, stained with an anti-CD3 mAb and fixed. Intracytoplamic staining with anti-Ki-67 mAb was performed using Perm/Wash kit (BD) according to the manufacturer’s instructions.

### Flow Cytometry

Fluorescein isothiocyanate, phycoerythrin, allophycocyanin, or Brilliant Violet 421 conjugated mAbs anti-CD1a, CD14, CD16, CD80, CD86, HLA-DR, CD83, CD206, CD1c, CD11b, DC-SIGN, CD4, CD3, CD39, CTLA-4, PD-1, Ki-67, IFN-γ, FoxP3, and CD25 were obtained from BD Pharmingen or BioLegend (Table 1). In all cases, isotype-matched control antibodies were used. Isotype staining and fluorescence minus one controls were performed using control DCs, DCs differentiated with PGE2 and with TGF-β (Figure S1 in Supplementary Material).

**Table 1:** List of antibodies used for flow cytometry.

	Brand	Clone	Fluorochrome	Catalog
CD1a	BD	HI149	APC	559775
CD1a	BD	HI149	FITC	555397
CD14	BD	M5E2	BV421	563743
CD14	BD	M5E2	FITC	555397
CD1c	BD	F10/21A3	PE	564900
CD11b	BD	ICRF44	PE	555388
HLA-DR	BD	L243	APC	340691
CD206	BD	19.2	PE	555954
DC-SIGN	BD	DCN46	APC	551545
CD16	BioLegend	3G8	APC	561248
CD80	BD	L307.4	FITC	557226
CD83	BD	HB15e	FITC	556910
CD86	BD	2331 (FUN-1)	PE	555658
CD40	BD	5C3	FITC	555588
CD4	BD	Leu-3a	BV510	562970
CD4	BioLegend	Leu-3	APC	357422
CD3	BD	SK7	APC-Cy7	557832
CD25	BD	M-A251	APC	555434
FoxP3	BD	259D/C7	Alexa 488	560047
CD39	BD	TU66	APC	560239
CTLA-4	BD	BNI3	PE	555853
PD-1	BD	EH12.1	PE-Cy7	561272
Ki-67	BioLegend	Ki-67	Alexa 488	350508
IFN-γ	BioLegend	4SB3	FITC	502506

*Fluorescein isothiocyanate (FITC), phycoerythrin (PE), phycoerythrin-Cy7 (PE-Cy7), allophycocyanin (APC), allophycocyanin-Cy7 (APC-Cy7), Brilliant Violet 421 (BV421), or Brilliant Violet 510 (BV510) conjugated mAbs anti-CD1a, CD14, CD16, CD80, CD86, HLA-DR, CD83, CD206, CD1c, CD11b, DC-SIGN, CD4, CD3, CD39, CTLA-4, PD-1, Ki-67, IFN-γ, FoxP3, and CD25 were obtained from BD Pharmingen or BioLegend*.

Analysis was performed by using a FACS Canto II flow cytometer (BD) and FlowJo X v10.0.7r2 software. The results are expressed as the mean fluorescence intensity (MFI), median fluorescence intensity, or as the percentage of positive cells.

### Fluorescence Microscopy

#### CD1a Staining

Dendritic cells were placed on poly-l-lysine-coated glass coverslips (12 mm) for 30 min at room temperature. Then, cells were washed, fixed in 4% paraformaldehyde (10 min on ice), and washed twice with 0.1 mM glycine in PBS. Next, cells were incubated with a mouse anti-CD1a mAb for 1 h, and then with Alexa 594-conjugated anti-mouse mAb (Jackson ImmunoResearch Laboratories) for 1 h.

#### Smad2/3 Staining

Monocytes were placed on poly-l-lysine-coated glass coverslips (12 mm) for 20 min at room temperature. Then, cells were washed, fixed in 4% paraformaldehyde (10 min on ice), and washed twice with 0.1 mM glycine in PBS. Subsequently, cells were incubated with a mouse mAb anti-human Smad2/3 (BD catalog 610843, recognizes total Smad2 and Smad3 regardless of phosphorylation) for 1 h, and then with Alexa 488-conjugated anti-mouse mAb (Jackson ImmunoResearch Laboratories) for 45 min.

In both cases, coverslips were mounted on glass slides using DAPI-Fluoromount G. Immunofluorescence images were acquired with an Eclipse Ti-S fluorescence microscope (Nikon) using an S Fluor Plan 40× objective and images were analyzed using the NIS-Elements Imaging software version 4.20 (Nikon).

### Quantitative Real-Time RT-PCR (Q-PCR)

Total RNA was obtained from 1 × 10^6^ cells using RNEasy kit (QIAGEN) and then treated with DNAse for 15 min (SIGMA). Reverse transcription was carried out using M-MLV reverse transcriptase (SIGMA) according to the manufacturer’s instructions. Briefly, 500 ng of RNA was incubated for 50 min at 37°C in the presence of 150 ng of random hexamer primers (Thermo Fisher), 0.01 M DTT, and 10 mM dNTP mix. Q-PCR was performed using Taqman Fast Master Mix (Thermo Fisher) in 10 µl reaction and Taqman gene expression kits, as follows: COX2 (Hs00153133_m1) and glyceraldehyde-3-phosphate dehydrogenase (GAPDH) (Hs99999905_m1). Reactions were carried out in a Step One Plus cycler (Thermo Fisher). The cycling program used was 95°C for 5 min followed by 40 cycles of 95°C for 5 s and 60°C for 30 s. Results are shown as 2^−ΔCt^ using GAPDH as a reference gene (40).

### Measurement of Cytokine Production

Supernatant harvested from DC cultures or mixed lymphocyte cultures were diluted with culture medium and the presence of IL-10, IL-12p70, IL-6, IL-23, IFN-γ, and IL-5 was assessed by ELISA (BD Biosciences), according to the manufacturer’s recommendations. For determination of intracytoplasmic IFN-γ expression, CD4+ T cells were coincubated with DCs for 4 days and then stained with anti-CD4 mAb and fixed. Intracytoplamic staining with anti-IFN-γ mAb was performed using Perm/Wash kit (BD) according to the manufacturer’s instructions.

### Western Blot

Nuclear and cytosolic protein fractions were prepared using NE-PER kit (Thermo Fisher) according to the manufacturer’s instructions. Briefly, cells were washed in PBS, then the plasma membrane was lysed and nuclei were separated from cytosolic protein fraction by centrifugation for 10 min at 12,000 *g*. Nuclear protein extract was obtained by lysis in NER buffer. Protein extracts were quantified by BCA assay and resuspended in 50 mM Tris–HCl pH 6.8, 2% SDS, 100 mM 2-mercaptoethanol, 10% glycerol, and 0.05% bromophenol blue. Protein extracts (20 µg) were electrophoresed on 8–15% SDS polyacrylamide gel and transferred to PVDF membranes. Blots were blocked with 5% non-fat powdered milk in TBS containing 0.05% Tween-20 and probed with the indicated primary antibodies followed by horseradish-peroxidase-conjugated secondary antibodies. Reactivity was developed by enhanced chemiluminescence according to the manufacturer’s instructions (Kalium Technologies).

Primary antibodies were mouse anti-human Smad2/3 (BD), mouse anti-human histone deacetylase-1 (Abcam catalog 68436), and rabbit anti-human GAPDH (Abcam catalog 22555). To augment sensitivity of Smad2/3 western blot, a goat anti-mouse secondary antibody was used followed by an anti-goat antibody conjugated to horseradish peroxidase.

### Statistics

Statistical significance was calculated using GraphPad software. Student’s paired *t*-test was used to determine the significance of differences between two treatment groups. For multiple analyses, one-way or two-way ANOVA were used followed by Bonferroni’s posttest for selected multiple comparisons. When tests for paired samples were used, it has been indicated in the figure legend. *p*-Values <0.05 were considered statistically significant, indicated by symbol “*”. Symbol “^#^” has been used to indicate *p* values <0.05 when comparing control DCs versus TGF-β-treated DCs.

## Results

**Figure 1:**
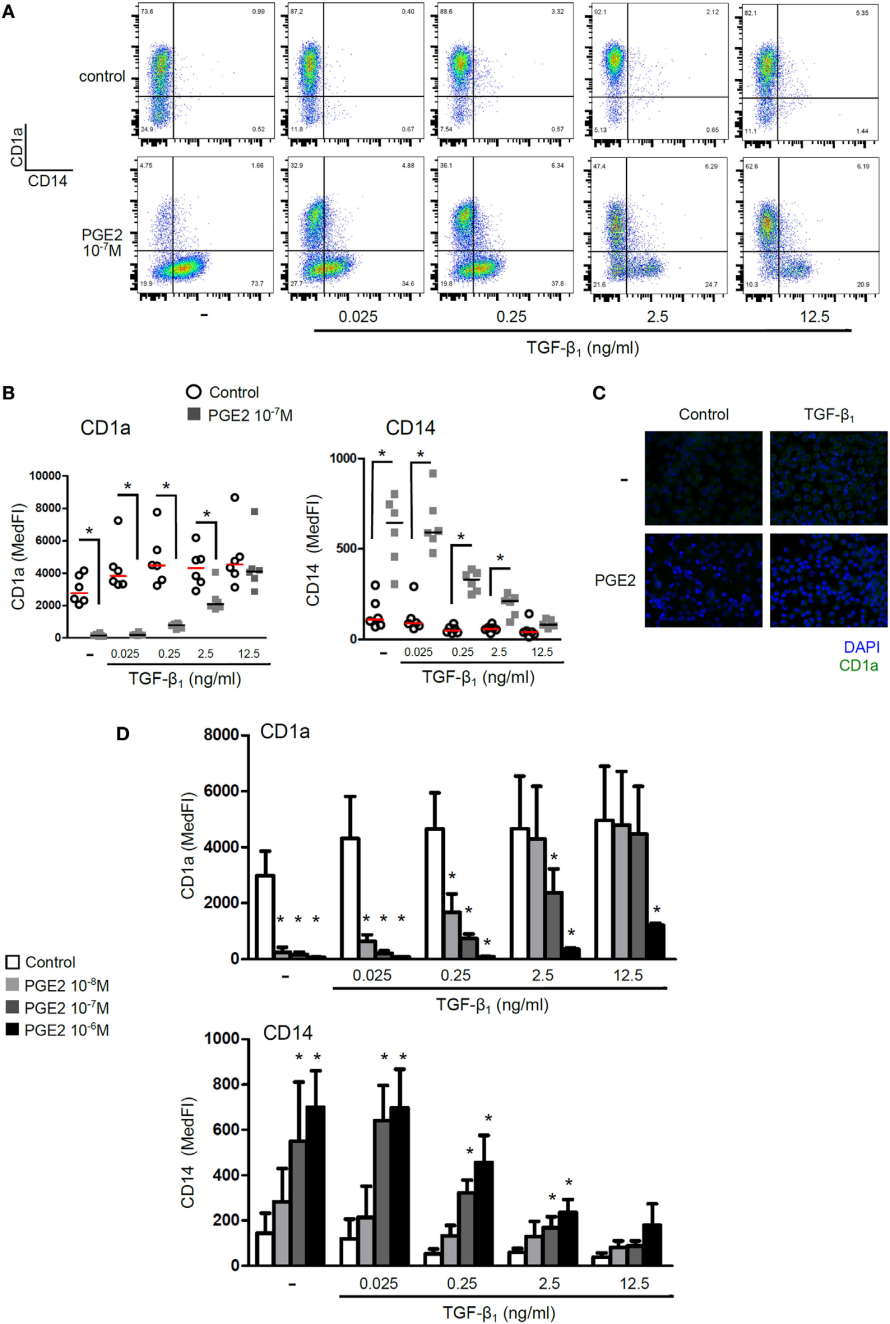
Presence of PGE2 during differentiation of DCs reverses the phenotype induced by TGF-β. Monocytes were incubated for 5 days with IL-4 and GM-CSF (control DCs), in the presence of TGF-β, PGE2, or a combination of both. Concentrations are as indicated. At day 5, expression of CD1a and CD14 was analyzed by flow cytometry. **(A)** Representative dot plots are shown. **(B)** Results from individual donors are expressed as MedFI with median bar. **(C)** Fluorescence microscopy of DCs stained with anti-CD1a and DAPI nuclear-staining. **(D)** Expression of CD1a and CD14 for different concentrations of PGE2 and TGF-β, as indicated. Bars indicate mean ± SEM of MedFI (*n* = 6). In all cases, * indicates *p* < 0.05 against control (i.e., condition without PGE2) as calculated after repeated-measures two-way ANOVA test, followed by Bonferroni posttest for selected comparisons. Abbreviations: MedFI, median fluorescence intensity; MFI, mean fluorescence intensity; PGE2, prostaglandin E2; DCs, dendritic cells.

In a first set of experiments, we analyzed the combined effect of PGE2 and TGF-β on the phenotype of DCs. Human monocytes were cultured with IL-4 and GM-CSF for 5 days in the presence of TGF-β, PGE2, or both. Analysis at day 5 showed that control DCs expressed high levels of CD1a and were negative for the expression of CD14 (Figure 1A). In agreement with previous reports, addition of TGF-β in the course of DC differentiation further increased CD1a expression (17), while cells incubated with PGE2 did not gain expression of CD1a and maintained CD14 expression (37, 41) (Figures 1A–C; Figure S2 in Supplementary Material). Analysis of DCs differentiated in the presence of both TGF-β and PGE2 revealed that PGE2 strongly antagonized the effects mediated by TGF-β in a concentration-dependent mode (Figures 1A–C). Remarkably, using concentrations of TGF-β similar to those found at inflammatory sites such as pleural effusions, synovial fluid, and tumor-derived ascites (~0.25 ng/ml) (42–46), we found that all concentrations of PGE2 assessed markedly suppressed the expression of CD1a, preserving CD14 expression (Figure 1D).

**Figure 2:**
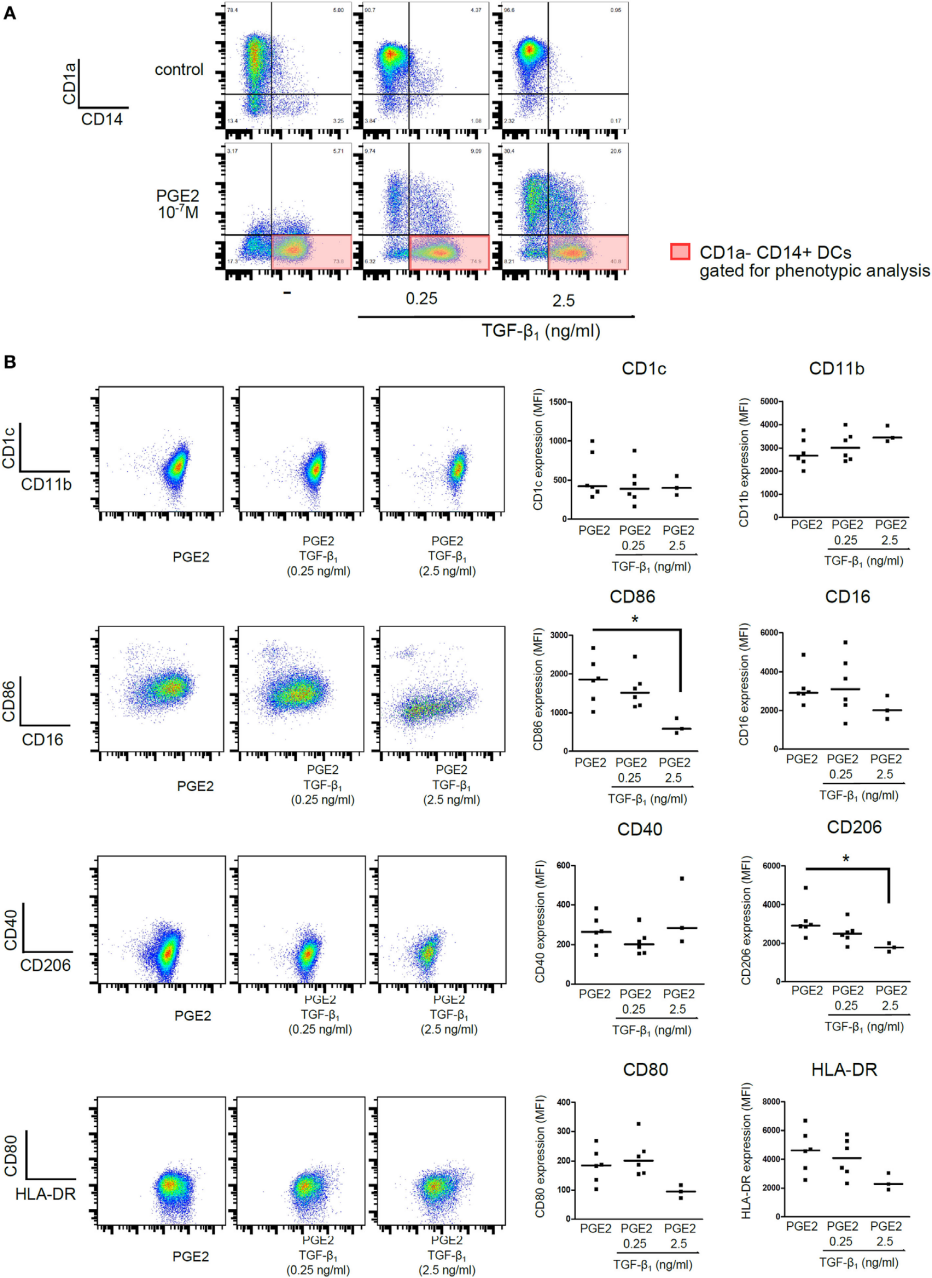
Presence of PGE2 during differentiation of DCs imposes a phenotypic signature, regardless of addition of TGF-β. Monocytes were incubated for 5 days with IL-4 and GM-CSF (control DCs), in the presence of TGF-β (0.25 and 2.5 ng/ml), PGE2 (10^−7^ M), or a combination of both. **(A)** Representative dot plots for expression of CD1a and CD14, showing gating of the CD1a− CD14+ subpopulation for further phenotypic analysis. **(B)**. Representative dot plots and quantification showing expression of selected markers, analyzed in the CD1a−CD14+ subpopulation. Results from individual donors are expressed as MFI with median bar (*n* = 3–6). * indicates *p* < 0.05 as calculated after one-way ANOVA test, followed by Bonferroni posttest for selected comparisons. Abbreviations: MFI, mean fluorescence intensity; PGE2, prostaglandin E2; DCs, dendritic cells.

Additional experiments were then performed by analyzing CD1a− CD14+ DCs (subpopulation gated as shown in Figure 2A) to define whether the phenotype obtained by differentiating DCs in the presence of PGE2 plus TGF-β was similar to the phenotype of those DCs differentiated in the presence of PGE2 alone. The following markers were analyzed: CD11b, CD1c, CD80, CD86, CD40, CD16, CD206, and HLA-DR. All these experiments were performed using TGF-β concentrations of 0.25 and 2.5 ng/ml and PGE2 concentration of 10^−7^ M. No differences in cell viability were observed between control and PGE2 plus TGF-β-treated cells (Figure S3 in Supplementary Material). Our results indicated that DCs differentiated in the presence of PGE2 plus TGF-β and those differentiated in the presence of PGE2 alone showed a similar phenotype signature. However, TGF-β concentrations of 2.5 ng/ml significantly reduced the expression of CD86 and CD206 (Figure 2B). For comparison, phenotypic analysis of CD1a+ CD14− DCs obtained in control and TGF-β-treated cultures is shown in Figure S4 in Supplementary Material.

**Figure 3:**
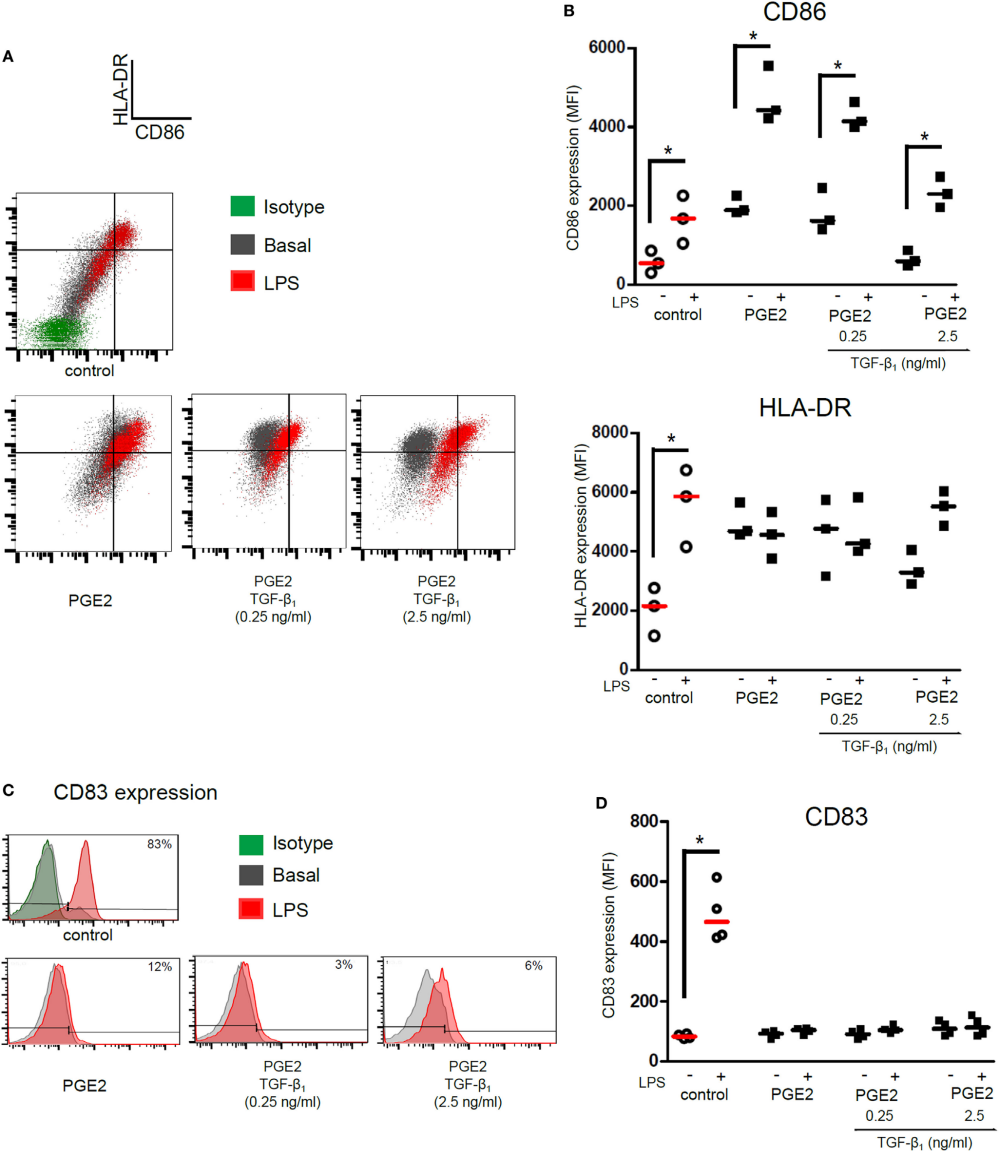
Presence of PGE2 during differentiation of DCs determines a phenotype signature upon LPS stimulation, regardless of addition of TGF-β. Monocytes were incubated for 5 days with IL-4 and GM-CSF in the presence of PGE2 (10^−7^ M), with or without addition of TGF-β (0.25 and 2.5 ng/ml). Next, DCs were washed and exposed for 24 h to LPS (20 ng/ml). Further analysis was performed in the gated CD1a− CD14+ subpopulation. **(A)** Representative dot plots for expression of CD86 and HLA-DR, analyzed in the CD1a− CD14+ subpopulation. **(B)** Results from individual donors are expressed as MFI with median bar (*n* = 3). **(C)** Representative histograms for expression of CD83, analyzed in the CD1a− CD14+ subpopulation. **(D)** Results from individual donors are expressed as MFI with median bar (*n* = 4). In all cases, * indicates *p* < 0.05 as calculated after repeated-measures one-way ANOVA test. Abbreviations: MFI, mean fluorescence intensity; PGE2, prostaglandin E2; DCs, dendritic cells.

We then analyzed the ability of DCs to acquire a mature phenotype upon stimulation with LPS. As expected, control DCs increased the expression of HLA-DR, CD86, and CD83 in response to LPS stimulation. DCs differentiated in the presence of PGE2 alone increased the expression of CD86 upon LPS stimulation, but not the expression of HLA-DR (Figures 3A,B). Expression of maturation marker CD83 was not be upregulated by LPS in these cells (Figures 3C,D). Interestingly, CD1a− CD14+ DCs differentiated in the presence of both PGE2 and TGF-β showed a similar response to LPS compared with those DCs differentiated in the presence of PGE2 alone (Figures 3A–D). In sharp contrast, DCs differentiated in the presence of TGF-β alone showed impaired expression of HLA-DR and CD86, but significantly increased the expression of CD83, upon LPS stimulation (Figure S5 in Supplementary Material), in agreement with previous reports (17, 18, 47). Taken together, these results suggest that PGE2 efficiently antagonizes the actions induced by TGF-β in terms of the LPS-induced phenotype of DCs.

**Figure 4:**
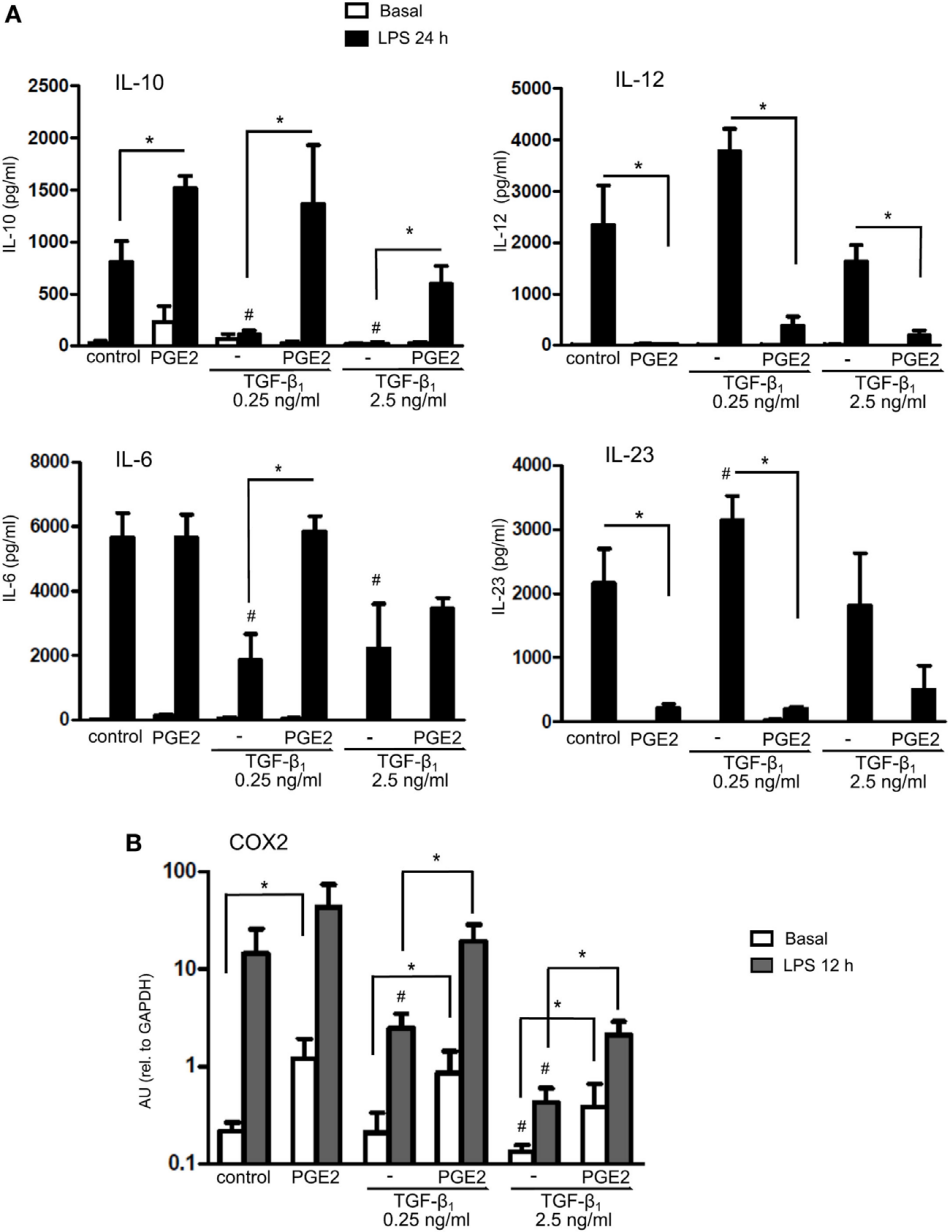
Presence of PGE2 during differentiation of DCs prevents TGF-β-mediated inhibition of IL-10 and COX2 expression. **(A)** Monocytes were incubated for 5 days with IL-4 and GM-CSF (control DCs), in the presence or absence of TGF-β (0.25 and 2.5 ng/ml), PGE2 (10^−7^ M), or a combination of both. Next, DCs were washed and exposed for 24 h to LPS (20 ng/ml). IL-10, IL-6, IL-12p70, and IL-23 were measured by ELISA in culture supernatants (mean ± SEM, *n* = 4–7). **(B)** Monocytes were incubated for 5 days with IL-4 and GM-CSF (control DCs), in the presence of TGF-β (0.25 ng/ml), PGE2 (10^−7^ M), or a combination of both. Next, DCs were washed and exposed for 12 h to LPS (20 ng/ml). COX2 was measured by q-PCR (mean ± SEM, *n* = 3–5). In all cases, * indicates *p* < 0.05 as calculated after one-way ANOVA test. ^#^ indicates *p* < 0.05 comparing TGF-β-treated versus control DCs. Abbreviations: AU, arbitrary units; PGE2, prostaglandin E2; DCs, dendritic cells.

Further studies were performed by studying the production of cytokines by LPS-stimulated DCs. Differentiation of DCs in the presence of PGE2 almost completely suppressed DC ability to produce IL-12 and IL-23, while increased the production of IL-10 without modifying the production of IL-6 compared with control DCs (Figure 4A). Conversely, differentiation of DCs in the presence of TGF-β substantially suppressed the production of IL-10 and IL-6. DCs differentiated in the presence of both TGF-β and PGE2 showed a pattern of cytokine production similar to DCs differentiated in the presence of PGE2 alone, although this effect was less marked when TGF-β was used at 2.5 ng/ml (Figure 4A), suggesting that antagonism of TGF-β by PGE2 is less efficient at a higher TGF-β concentration (in agreement with data from CD1a and CD14 expression as shown in Figure 1). Furthermore, we studied the expression of prostaglandin-endoperoxide synthase-2 [cyclooxygenase-2 (COX2)], since PGE2 has been reported to redirect DCs toward a tolerogenic phenotype by inducing COX2 expression and triggering a positive feedback loop (25, 43). We confirmed that PGE2 increased COX2 expression compared to control DCs (Figure 4B). We also found that TGF-β-treated DCs reduced LPS-induced expression of COX2 compared to control, whereas the addition of PGE2 reversed this inhibition, again supporting the conclusion that PGE2 antagonizes the effect of TGF-β.

**Figure 5:**
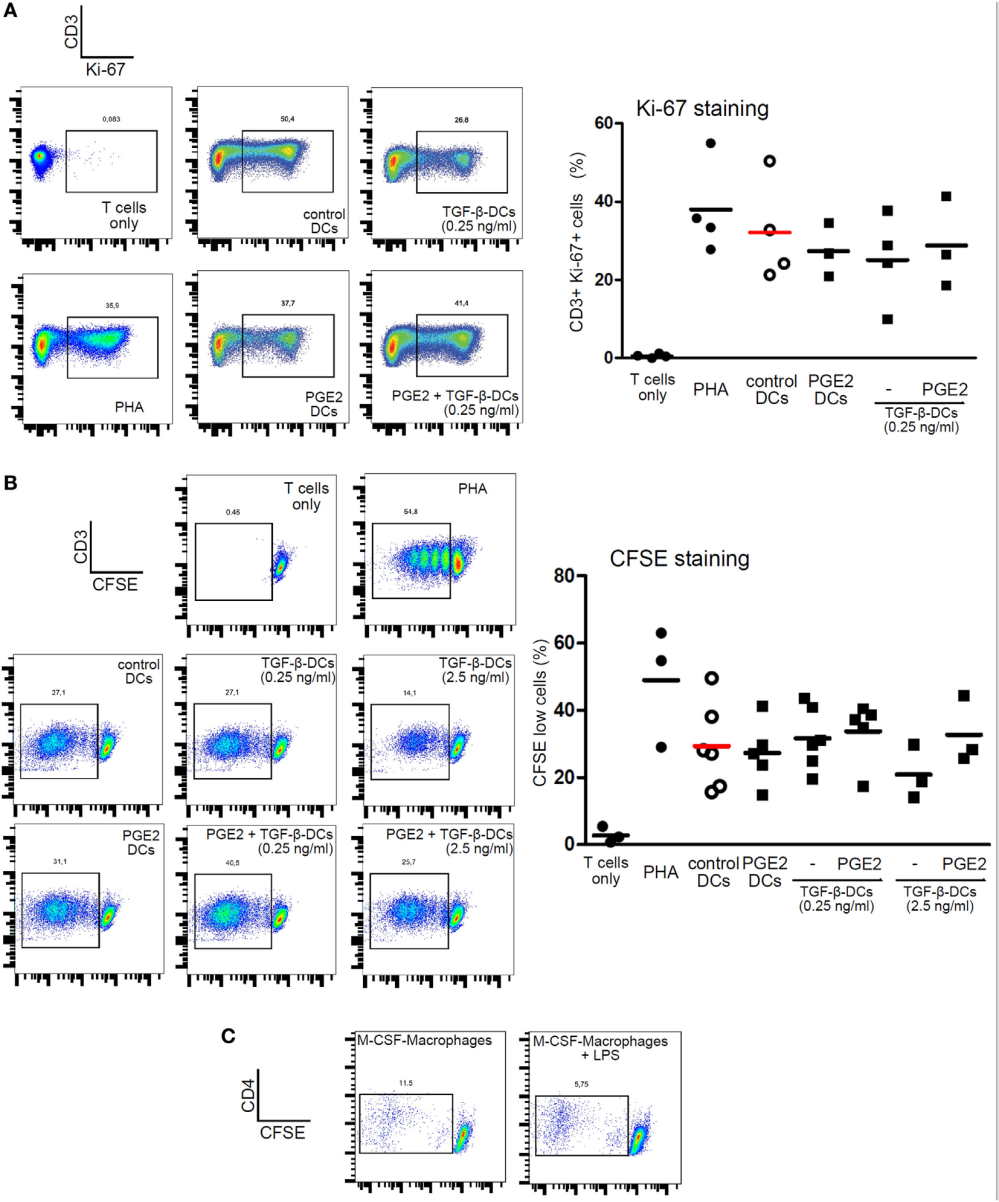
Dendritic cells (DCs) differentiated in the presence of prostaglandin E2 (PGE2), TGF-β, or a combination of both are all equally capable of inducing proliferation of allogeneic lymphocytes. Monocytes were incubated for 5 days with IL-4 and GM-CSF (control DCs), in the presence of TGF-β (0.25 and 2.5 ng/ml), PGE2 (10^−7^ M), or a combination of both. Next, DCs were washed and exposed for 24 h to LPS (20 ng/ml). **(A)** LPS-stimulated DCs were washed and incubated with allogeneic CD4+ T cells using a DC:lymphocyte ratio of 1:4. At day 3, cells were harvested and the expression of Ki-67 was analyzed by intracellular staining and flow cytometry in the gate of CD3+ T cells. Representative dot plots are shown. Results from individual donors are expressed as % of Ki-67+ cells with mean bar (*n* = 3–4). **(B)** LPS-stimulated DCs were washed and incubated with CFSE-labeled allogeneic CD4+ T cells, using a DC:lymphocyte ratio of 1:4. At day 4, the dilution of CFSE staining in the gate of CD3+ T cells was measured by flow cytometry. Lymphocytes cultured alone with or without PHA (10 µg/ml) were used as positive and negative controls, respectively. Representative dot plots are shown. Results from individual donors are expressed as % of CFSE-low cells with mean bar (*n* = 3–6). **(C)** Monocytes were incubated for 7 days with M-CSF. Next, macrophages were washed and exposed, or not, for 24 h to LPS (50 ng/ml). Macrophages were washed and incubated with allogeneic CD4+ T cells using a macrophage:lymphocyte ratio of 1:4. The proliferative response of T cells was analyzed at day 4 by measuring the dilution of CFSE staining in the gate of CD3+ T cells by flow cytometry. Representative dot plots are shown (*n* = 3).

**Figure 6:**
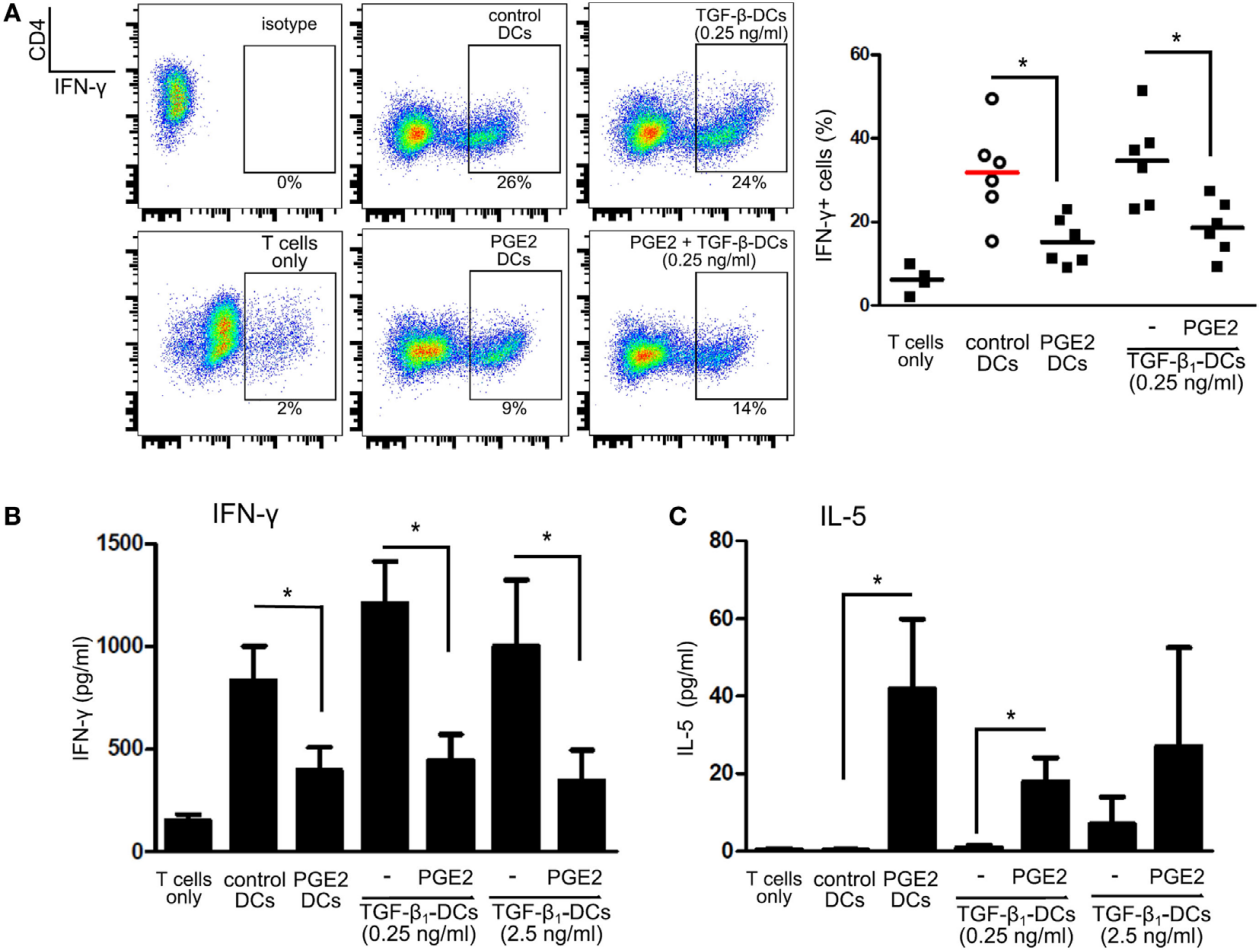
Prostaglandin E2 (PGE2) redirects differentiation of dendritic cells (DCs) suppressing IFN-γ production in mixed lymphocyte cultures, regardless of TGF-β addition. Monocytes were incubated for 5 days with IL-4 and GM-CSF (control DCs), in the presence of TGF-β (0.25 and 2.5 ng/ml), PGE2 (10^−7^ M), or a combination of both. Next, DCs were washed and exposed for 24 h to LPS (20 ng/ml). **(A)** LPS-stimulated DCs were washed and incubated with allogeneic CD4+ T cells using a DC:lymphocyte ratio of 1:4. At day 4, cells were harvested and the expression of IFN-γ was analyzed by intracellular staining and flow cytometry in the gate of CD4+ T cells. Representative dot plots are shown. Results from individual donors are expressed as % of IFN-γ+ cells with mean bar (*n* = 4–6). * indicates *p* < 0.05 as calculated after repeated-measures one-way ANOVA test, followed by Bonferroni posttest for selected comparisons. **(B)** IFN-γ and **(C)** IL-5 were measured by ELISA in culture supernatants at day 4 (mean ± SEM, *n* = 4–7). * indicates *p* < 0.05 as calculated after one-way ANOVA test.

**Figure 7:**
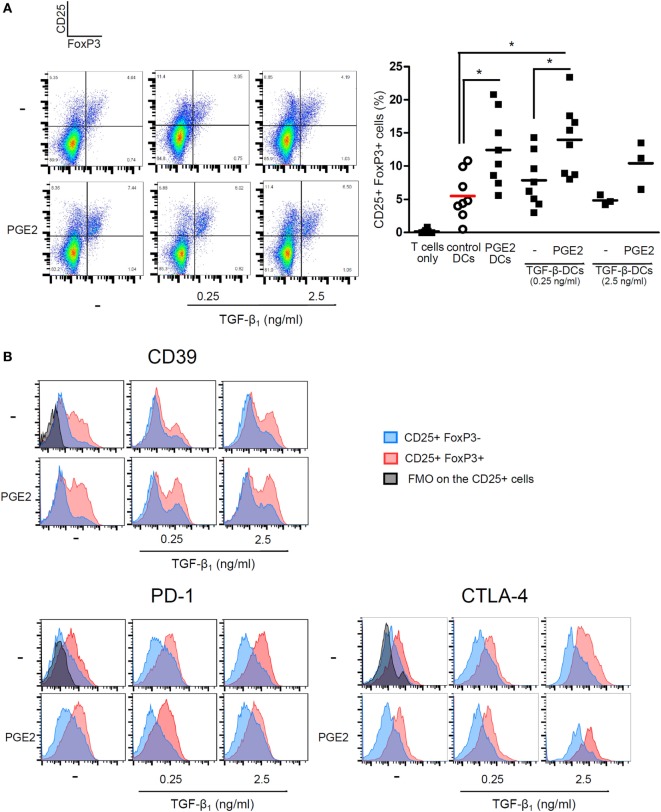
PGE2 redirects differentiation of DCs promoting the expansion of CD25+ FoxP3+ T cells, regardless of TGF-β addition. Monocytes were incubated for 5 days with IL-4 and GM-CSF (control DCs), in the presence of TGF-β (0.25 and 2.5 ng/ml), PGE2 (10^−7^ M), or a combination of both. Next, DCs were washed and exposed for 24 h to LPS (20 ng/ml). LPS-stimulated DCs were washed and incubated with allogeneic CD4+ T cells using a DC:lymphocyte ratio of 1:4 for 4 days. **(A)** Representative dot plots for expression of CD25 and FoxP3, analyzed in the CD4+ subpopulation. Results from individual donors are expressed as % of double positive CD25+ FoxP3+ cells with mean bar (*n* = 3–8). * indicates *p* < 0.05 as calculated after one-way ANOVA test, followed by Bonferroni posttest for selected comparisons. **(B)** Representative histograms for expression of CD39, PD-1, and intracytoplasmic CTLA-4, analyzed in the CD25+ FoxP3− and the CD25+ FoxP3+ subpopulations (*n* = 3). Abbreviations: FMO, fluorescence minus one controls; PGE2, prostaglandin E2; DCs, dendritic cells.

We then analyzed the ability of LPS-stimulated DCs differentiated with TGF-β, PGE2, or both, to induce T cell proliferation by co-culturing DCs with allogeneic lymphocytes. Cell proliferation was analyzed by measuring either Ki-67 intracellular staining or CFSE dilution. All the different DC populations showed a high allostimulatory activity and no differences were observed among them (Figures 5A,B). This observation suggests that monocytes differentiated with GM-CSF plus IL-4, in the presence of PGE2 or PGE2 plus TGF-β, actually function as DCs in terms of their allostimulatory activity. This contrasts with the observations made with macrophages obtained from monocytes treated with M-CSF, which showed a very poor ability to stimulate the proliferation of allogeneic lymphocytes (Figure 5C). In spite of their similar allostimulatory activity, the different DC populations stimulated the production of cytokines by lymphocytes in a distinct fashion. Control DCs as well as DCs differentiated in the presence of TGF-β strongly stimulated the production of IFN-γ by allogeneic lymphocytes. A significant lower production of IFN-γ, however, was observed using DCs differentiated in the presence of PGE2 or PGE2 plus TGF-β (Figures 6A,B). By contrast, IL-5 production was undetectable in cultures prepared with control DCs and DCs differentiated in the presence of TGF-β, while an increased production of IL-5 was observed in cultures performed with DCs differentiated in the presence of PGE2 or PGE2 plus TGF-β (Figure 6C). On the other hand, analysis of the frequency of CD4+ CD25+ FOXP3+ T cells in the course of the mixture lymphocyte reaction revealed that cultures prepared with DCs differentiated in the presence of PGE2 or PGE2 plus TGF-β showed a higher frequency of this cell population (Figure 7A; Figure S6 in Supplementary Material). Supporting that CD4+ CD25+ FOXP3+ T cells are actually regulatory T cells, we found that these cells expressed higher levels of the T regulatory cell-makers CD39, PD-1, and intracellular CTLA-4 compared with CD4+ CD25+ FOXP3− T cells (Figure 7B). We conclude from these results that DCs differentiated in the presence of PGE2 and TGF-β, like DCs differentiated in the presence of PGE2 alone, prevent the activation of CD4+ T cells in a pro-inflammatory profile, promoting a regulatory signature.

**Figure 8:**
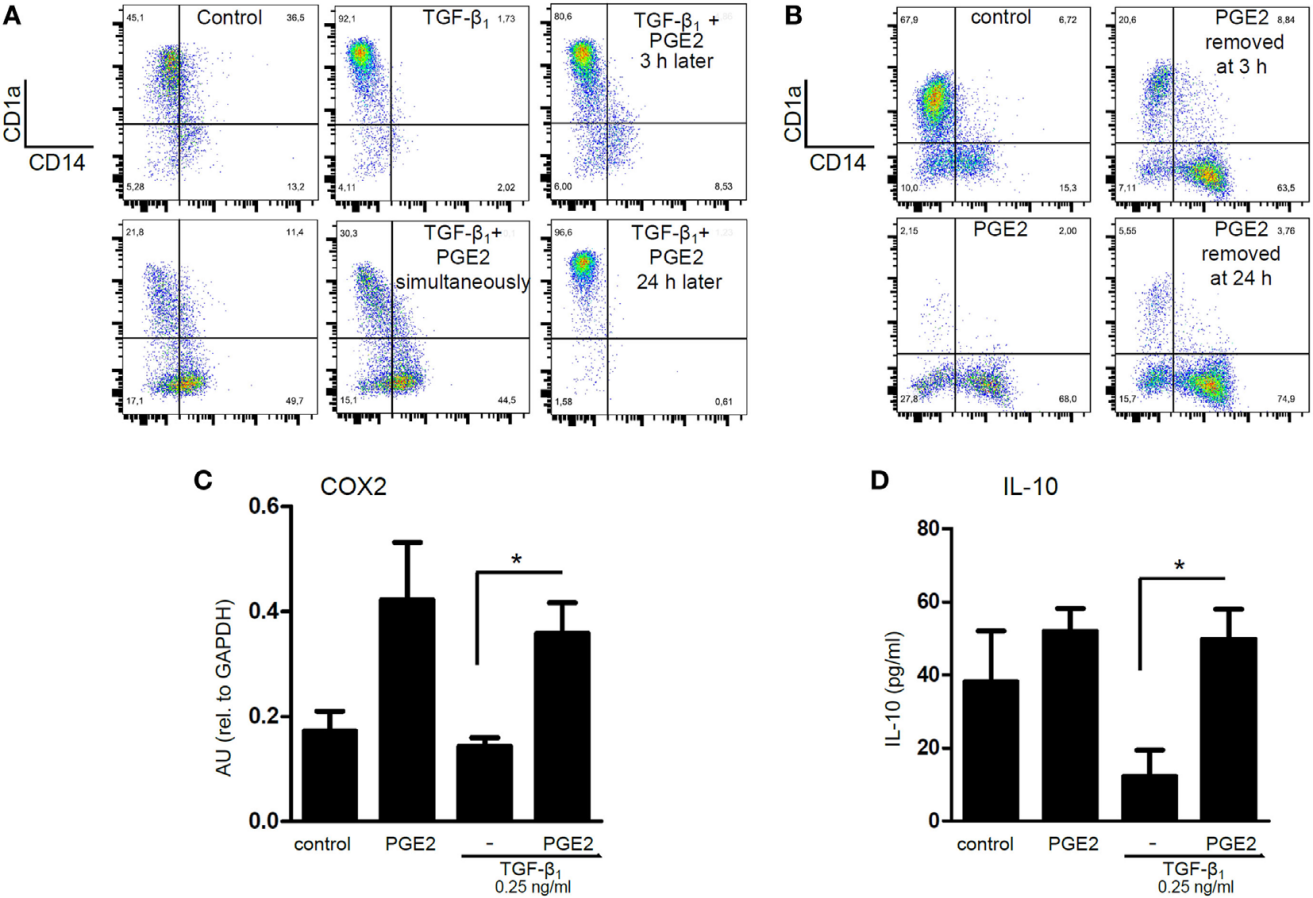
Prostaglandin E2 (PGE2) interferes with TGF-β actions acting at early time points after the onset of differentiation. **(A)** Monocytes were incubated with IL-4 and GM-CSF [control dendritic cells (DCs)], in the presence of TGF-β (0.25 ng/ml), PGE2 (10^−7^ M), or a combination of both. When indicated, PGE2 was added 3 and 24 h after the addition of TGF-β. At day 5, expression of CD1a and CD14 was analyzed by flow cytometry. Representative dot plots are shown (*n* = 3). **(B)** Monocytes were incubated with IL-4 and GM-CSF (control DCs), in the absence or in the presence of PGE2 (10^−7^ M). Cells were washed after 3 or 24 h of culture, and cultured in medium with IL-4 and GM-CSF without PGE2. At day 5, expression of CD1a and CD14 was analyzed by flow cytometry. Representative dot plots are shown (*n* = 3). **(C)** Monocytes were incubated as in **(A)**, but total RNA was extracted at 5 h of culture. COX2 was measured by q-PCR (mean ± SEM, *n* = 4). Abbreviation: AU, arbitrary units. **(D)** Monocytes were incubated as in **(A)**, but IL-10 was measured at 24 h by ELISA in culture supernatants (mean ± SEM, *n* = 6). In all cases, * indicates *p* < 0.05 as calculated after one-way ANOVA test, followed by Bonferroni posttest for selected comparisons.

**Figure 9:**
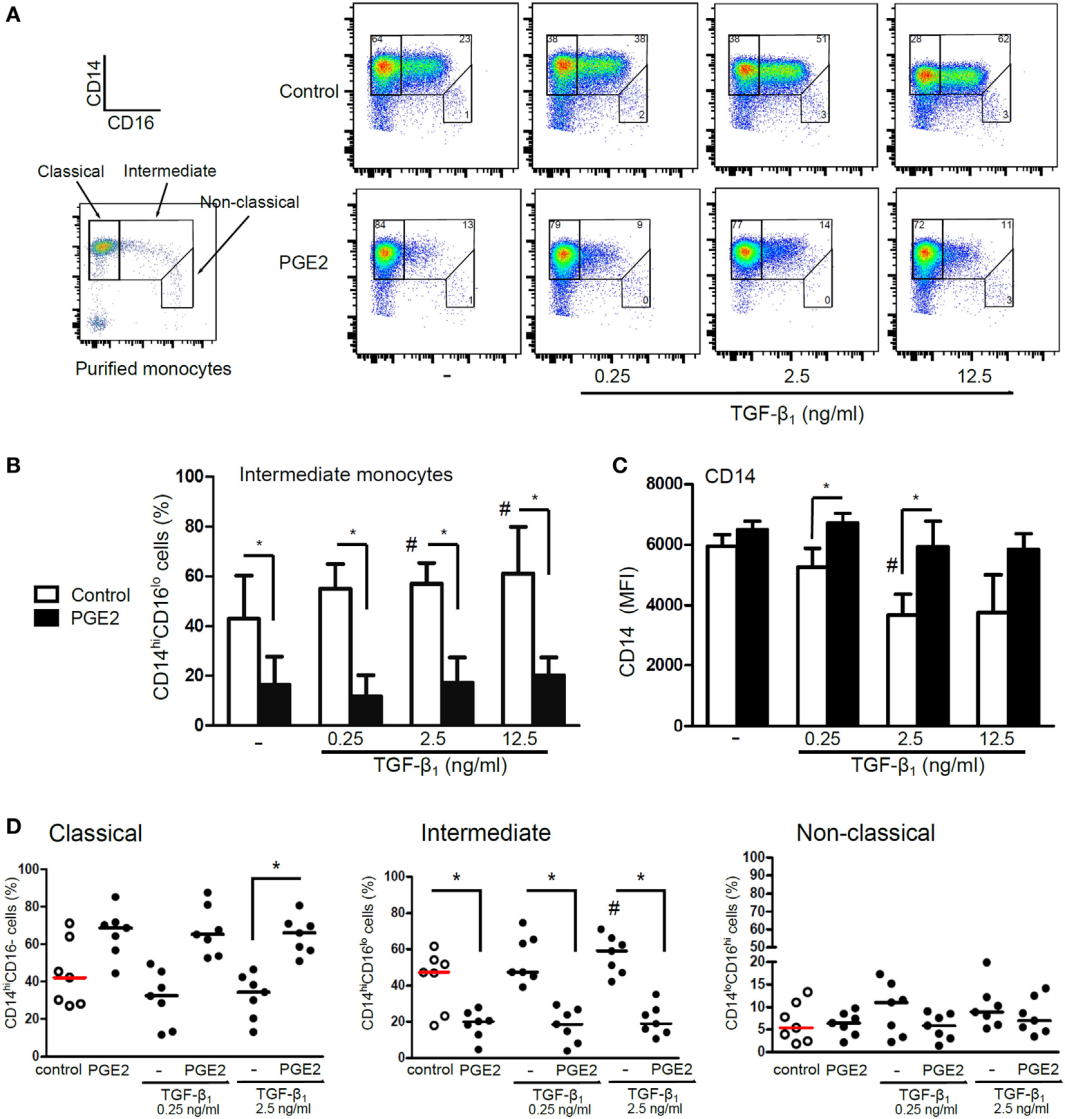
Presence of PGE2 antagonizes the induction of CD16 expression mediated by TGF-β. **(A–D)** Monocytes were obtained using CD14 microbeads and then incubated with IL-4 and GM-CSF (control) or with addition of TGF-β (concentrations as indicated), PGE2 (10^−7^ M), or a combination of both. At 24 h, expression of CD14 and CD16 was analyzed by flow cytometry and classical, intermediate, and non-classical monocyte subsets were defined as shown. **(A)** Representative dot plots are shown. **(B)** Quantification of intermediate monocyte subset (mean ± SEM, *n* = 4). **(C)** Expression of CD14 in the whole monocyte population (mean ± SEM, *n* = 4). **(D)** Quantification of monocyte subsets (*n* = 7). Results from individual donors are expressed as % of cells with median bar, obtained from cultures using TGF-β at 0.25 and 2.5 ng/ml. In all cases, * indicates *p* < 0.05 as calculated after repeated-measures two-way ANOVA test, followed by Bonferroni posttest for selected comparisons. ^#^ indicates *p* < 0.05 comparing TGF-β versus control DCs. Abbreviations: MFI, mean fluorescence intensity; PGE2, prostaglandin E2; DCs, dendritic cells.

We performed additional experiments to analyze the mechanisms through which PGE2 antagonized the actions induced by TGF-β in the course of DC differentiation. Analysis of DC phenotype performed at 5 days of culture revealed that the ability of TGF-β to increase CD1a expression was only modestly inhibited when PGE2 was added 3 h after the addition of TGF-β, while addition of PGE2 24 h later had no effect at all (Figure 8A). In agreement with this finding, removing PGE2 at either 3 or 24 h after the culture start did not impair the acquisition of a CD1a− CD14+ phenotype by DCs (Figure 8B). Consistent with these observations, we found that the ability of PGE2 to stimulate COX2 expression and IL-10 production was induced at early time points, even in the presence of TGF-β (Figures 8C,D). Taking these observations into account, we decided to analyze the changes in the expression of CD14 and CD16 by monocytes at 24 h of culture. At this time point, monocytes cultured with GM-CSF plus IL-4 did not express CD1a (data not shown), and showed an increased frequency of CD14hi CD16int cells [defined as intermediate monocytes (48)], compared with freshly isolated monocytes. The acquisition of this phenotype signature was further increased by TGF-β and was strongly prevented by PGE2 (Figures 9A–D). Together, these observations suggest that PGE2 antagonizes the effects induced by TGF-β on DC differentiation acting at early time points.

**Figure 10:**
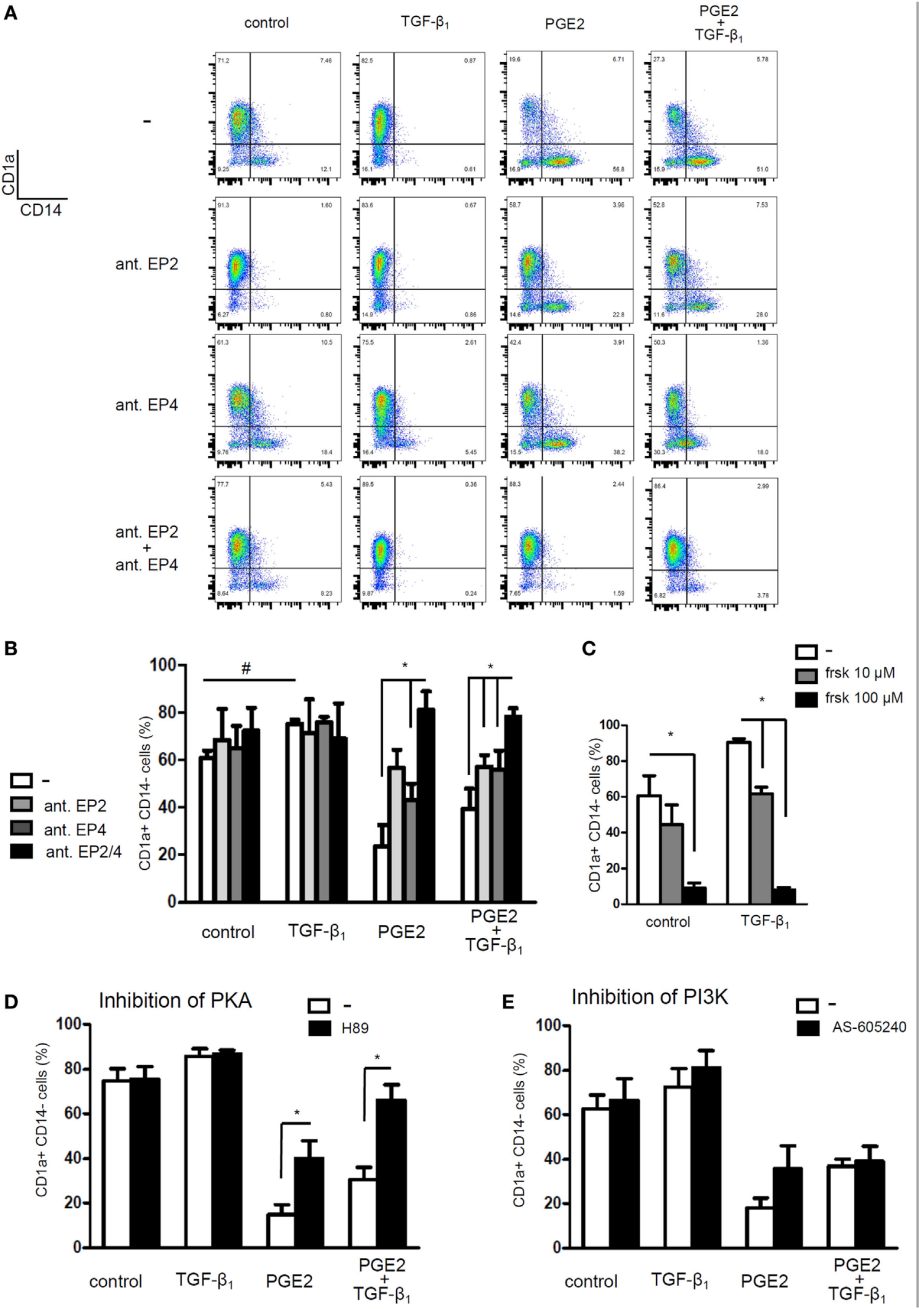
Prostaglandin E2 (PGE2) interferes with TGF-β actions by stimulating the increase in intracellular cyclic adenosine mono-phosphate increase and protein kinase A (PKA) activity. **(A,B)** Monocytes were pre-incubated for 20 min with EP2 receptor antagonist PF-04418948 (25 µM), EP4 receptor antagonist L-161,982 (25 µM), or both. Then, cells were cultured with IL-4 and GM-CSF, in the absence or presence of TGF-β (0.25 ng/ml), PGE2 (10^−7^ M), or a combination of both. At 24 h, cells were washed and cultured in medium with IL-4 and GM-CSF without PGE2 or TGF-β. At day 5, expression of CD1a and CD14 was analyzed by flow cytometry (mean ± SEM, *n* = 4–6). Representative dot plots are shown in **(A)**. **(C)** Monocytes were incubated with IL-4 and GM-CSF, in the absence or presence of TGF-β (0.25 ng/ml), the adenylate cyclase activator forskolin (10 and 100 µM), or a combination of both. At day 5, expression of CD1a and CD14 was analyzed by flow cytometry (mean ± SEM, *n* = 3). **(D,E)** Monocytes were pre-incubated for 1 h with **(D)** PKA inhibitor H89 (10 µM) or **(E)** phosphatidylinositol-3-phosphate kinase (PI3K) inhibitor AS-605240 (10 µM) before differentiation with IL-4 and GM-CSF, performed in the absence or presence of TGF-β (0.25 ng/ml), PGE2 (10^−7^ M), or a combination of both. At 24 h, cells were washed and incubated without antagonists. At day 5, expression of CD1a and CD14 was analyzed by flow cytometry (mean ± SEM, *n* = 4–5). In all cases, * indicates *p* < 0.05 as calculated after repeated-measures two-way ANOVA test, followed by Bonferroni posttest for selected comparisons. ^#^ indicates *p* < 0.05 comparing TGF-β-treated dendritic cells (DCs) versus control DCs.

Finally, we looked for possible mechanisms explaining the interaction of PGE2 and TGF-β signaling pathways. Monocytes express only two out of four E-prostanoid receptors, namely EP2 and EP4 (49, 50). We used EP2 receptor antagonist PF-04418948 (25 µM) and EP4 receptor antagonist L-161,982 (25 µM). Blocking EP2 or EP4 receptors separately only partially prevented the effect induced by PGE2. By contrast, it was completely prevented by incubating monocytes with both antagonists (Figures 10A,B). We speculated that a shared signaling cascade between the two EP receptors could be responsible for the inhibition of TGF-β actions. Since both EP2 and EP4 are coupled to an increase in intracellular cAMP, we hypothesized that this pathway could be responsible for antagonizing TGF-β signaling.

**Figure 11:**
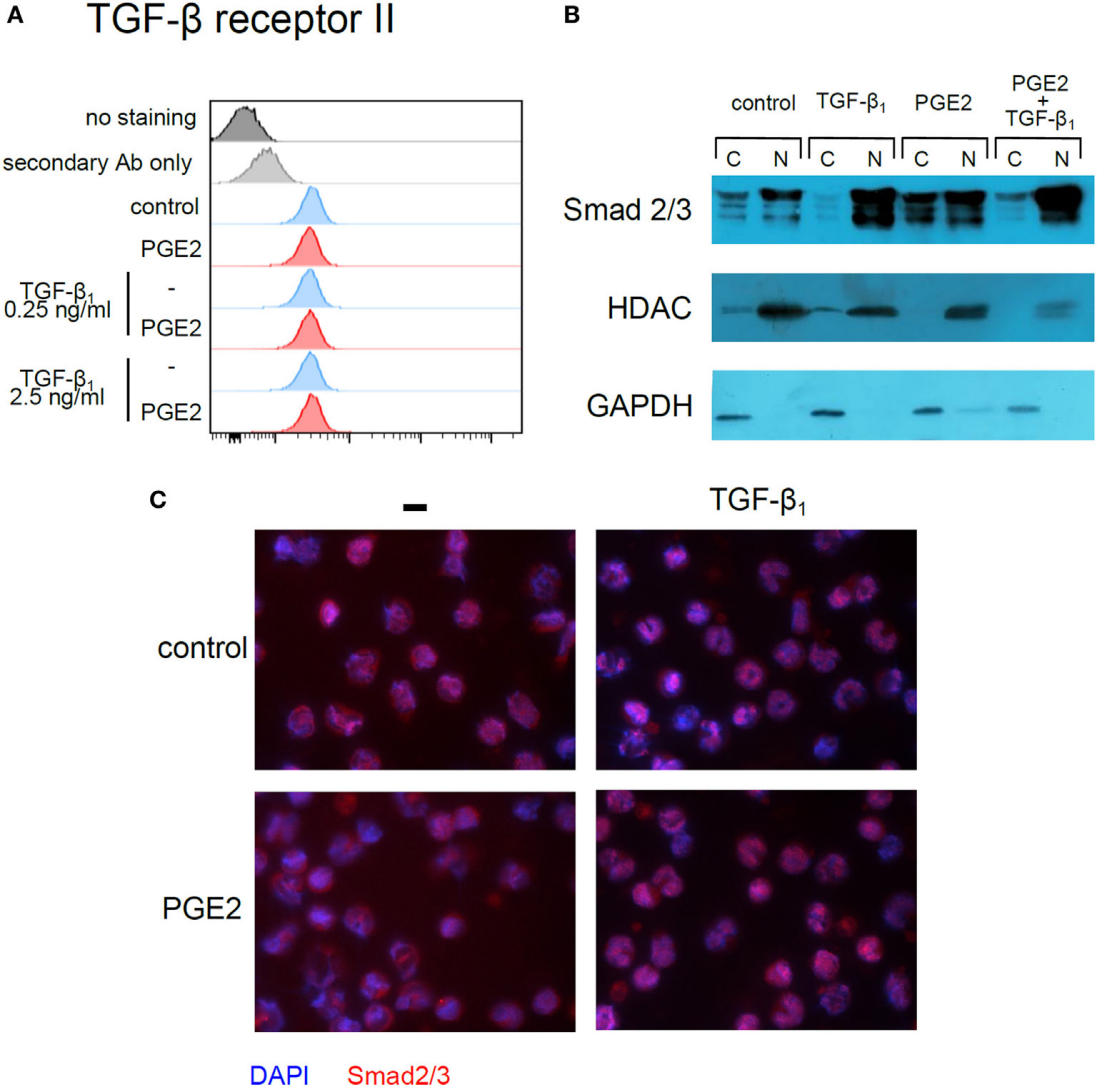
Prostaglandin E2 (PGE2) interferes neither with TGF-β receptor type II expression nor with TGF-β-induced Smad2/3 entry into the nucleus. **(A)** Monocytes were incubated with IL-4 and GM-CSF (control), in the absence or presence of TGF-β (0.25 and 2.5 ng/ml), PGE2 (10^−7^ M), or a combination of both. At 3 h, expression of TGF-β receptor type II was analyzed by flow cytometry (representative histograms shown, *n* = 3). Also shown are isotype staining control and staining only with secondary antibody (Alexa-594-conjugated anti-mouse). **(B,C)** Monocytes were incubated for 45 min with IL-4 and GM-CSF, in the absence or presence of TGF-β (1 ng/ml), PGE2 (10^−7^ M), or a combination of both. In **(B)**, cytoplasmic and nuclear extracts were prepared and total Smad2/3 was analyzed by western blot. Histone deacetylase-1 (HDAC1) and glyceraldehyde-3-phosphate dehydrogenase (GAPDH) were used as nuclear and cytoplasmic markers, respectively. Representative blots from *n* = 3 are shown. N, nuclear extract; C, cytoplasmic extract. In **(C)**, cells were fixed at 45 min and stained with anti-Smad2/3 for fluorescence microscopy. Representative photographs from *n* = 3 are shown.

Previous studies in fibroblasts have demonstrated that a cAMP increase, followed by PKA-induced phosphorylation of cAMP-responsive-element binding protein (CREB), leads to sequestration of transcriptional coactivator CBP/p300, also involved in Smad-mediated transcription, and consequently to weakening of TGF-β signaling (38). To evaluate this mechanism, we first tested whether forskolin, an adenylyl cyclase activator, mimicked the effect of PGE2. In fact, monocyte differentiation in the presence of forskolin (10 and 100 µM) resulted in CD1a− CD14+ DCs, regardless of the presence of TGF-β (Figure 10C) and thus confirmed that cAMP elevation counteracts TGF-β activity. Accordingly, the PKA inhibitor H-89 (10 µM) partially prevented the effect induced by PGE2, either in the absence or presence of TGF-β (Figure 10D), further supporting a role of the cAMP–PKA pathway in the actions exerted by PGE2 (Figure 10D). By contrast, inhibition of EP4-activated PIK3γ by treatment with AS-605240 (10 µM) did not prevent PGE2 actions (Figure 10E). On the other hand, results in Figure 11A shows that differentiation of DCs in the presence of PGE2 did not result in the inhibition of TGF-β receptor type II expression. In addition, we studied the cellular localization of Smad2 and Smad3 after treatment of monocytes with TGF-β, PGE2, or both. Smad2 and Smad3 are two transcription factors that localize to the nucleus when phosphorylated as part of the canonical TGF-β-signaling cascade (9). We analyzed total Smad2/3 cellular localization by western blot and fluorescence microscopy and confirmed that PGE2 did not interfere with TGF-β-induced translocation of Smad2/3 into the nucleus (Figures 11B,C). Altogether, our results indicate that PGE2 interference of TGF-β signaling is mediated through elevation of cAMP and PKA activity, without compromising Smad2/3 activation by TGF-β. Interestingly, a similar mechanism through which PGE2 antagonizes the effect of TGF-β has been previously described in fibroblasts (38).

## Discussion

Dendritic cells are a heterogeneous group of cells that share strong antigen-presenting capabilities, but differ in their ontogeny, phenotype, and other functional characteristics. In the steady state, DCs resident in lymphoid organs as well as migratory DCs populating peripheral tissues originate from circulating committed precursors. Thus, in non-inflammatory conditions, most populations of DCs are replenished from these precursors without requirement of monocyte input from blood (51). By contrast, during pathogen-induced or sterile inflammation, blood monocytes infiltrate the inflammation site and give raise to a distinct subset of DCs termed inflammatory DCs.

In mouse, the relevance of monocyte-derived inflammatory DCs has been established for several years (52). For instance, they have been proven necessary for protective Th1 responses against *Leishmania major*, HSV-2, or influenza virus (2, 52, 53), as well as Th2 responses (4, 54) and even for the prevention of exacerbated local innate responses after *Toxoplasma gondii* infection (6). In humans, characterization of inflammatory DCs has lagged behind, but still they have been described in synovial fluid from rheumatoid arthritis patients, tumor-derived ascites and skin from psoriasis and atopic dermatitis patients (1, 55, 56), where they have also been linked to different profiles of the immune response.

From these studies, it becomes clear that infiltrating monocytes are highly plastic cells. In this regard, a comprehensive transcriptional profiling study demonstrated that the functional properties of tissue-resident DCs (from skin and lungs) are defined mainly by their ontogeny, whereas the profile acquired by monocyte-derived inflammatory DCs is rather dependent on microenvironmental signals (7). In the same sense, two recent studies using single-cell RNA-seq transcriptomics have shown homogeneous gene expression signatures within the blood CD14+ monocytes subset (57, 58), thus indicating that monocytes are not predetermined but instead rely on external signals to define their fate. For instance, Goudot et al. observed that a uniform population of CD14+ monocytes could switch their transcriptional program to differentiate either to DCs or macrophages (58). Interestingly, the proportion of one cell type over the other correlated dose-dependently with the presence of ligands to the aryl hydrocarbon receptor, providing a hint to devise the environmental signals involved in the differentiation of inflammatory DCs. However, the factors affecting the differentiation of monocytes to DCs *in vivo* still remain largely unknown.

TGF-β and PGE2 are two ubiquitous immunomodulators known to coexist in inflammatory sites and the tumor microenvironment (59, 60), where differentiation of DCs from monocytes takes place. Here, we focused on the interaction of these two mediators acting on monocytes during their differentiation to DCs, as opposed to studying their actions on fully differentiated DCs. A review of the literature revealed that key properties of DCs, most notably the cytokine expression pattern, are modulated in opposite ways by the presence of TGF-β and PGE2 during differentiation (17, 21). In this work, we studied how these two factors affected the outcome of DC differentiation when acting together. Using the well-known model of DC differentiation induced by IL-4 and GM-CSF (61), we found that incubation with PGE2 promoted IL-10-producing DCs which induced expansion of CD25+ FoxP3+ T cells, whereas incubation with TGF-β blocked IL-10 but enabled the differentiation of IL-12-producing DCs capable of stimulating Th1 responses. Remarkably, when we added PGE2 and TGF-β together at physiologically relevant concentrations, we found that PGE2 overcame the influence of TGF-β, leading to DCs that produced high levels of IL-10 but not IL-12, upregulated the expression of COX2, did not mature completely in response to LPS and promoted the expansion of regulatory T lymphocytes. Previous studies demonstrated that PGE2 is linked to the *in vitro* and *in vivo* development of MDSCs (25, 62). In this context, our results further underscore the relevance of PGE2 as an immunosuppressive factor acting on the differentiation of DCs. What is more, our data showed that antagonizing TGF-β required the presence of PGE2 only at the beginning of the differentiation process, thus opening the possibility that short spikes of PGE2 levels could instruct monocytes to disregard longer TGF-β signals. Interestingly, this temporal succession has actually been described in a murine model of acute peritonitis followed by a resolution period (63). In summary, we conclude that the presence of PGE2 is likely to minimize the effects mediated by TGF-β on the differentiation of DCs.

These results are consistent with our previous report investigating the differentiation of DCs in the presence of seminal plasma, a fluid extremely rich in TGF-β and prostanoids of the E series, including PGE1, PGE2, and their 19-OH-derivatives (37). Deposition of semen triggers an inflammatory reaction involving secretion of CCL2, IL-8, and GM-CSF by epithelial cells, which leads to a rapid influx of CD14+ monocytes to the genital mucosa (64). In this context, inflammatory monocytes may be exposed to seminal TGF-β1, averaging 200 ng/ml, as well as PGE prostanoids which can reach values in semen as high as 10^−3^M (35, 36, 65). By incubating monocytes in the presence of seminal plasma, together with IL-4 and GM-CSF, we obtained CD1a− CD14+ DCs with enhanced production of IL-10 that promoted the expansion of FoxP3+ regulatory T cells (37). Notably, antagonizing receptors EP2 and EP4 abrogated the effect induced by seminal plasma and resulted in DCs bearing the typical CD1a+ CD14− phenotype. We concluded that seminal plasma prostaglandins were responsible for the generation of tolerogenic DCs, thus providing a possible mechanism for previous observations indicating that exposure to semen, even in the absence of embryo implantation, was associated with the expansion of regulatory T cells specific to paternal antigens (66, 67). Therefore, our previous results together with the present work indicate that TGF-β does not play a critical role in promoting a tolerogenic profile in DCs that survey paternal antigens. On the other hand, Sharkey et al. elegantly demonstrated that seminal TGF-β induced the secretion of IL-6, CCL2, and GM-CSF from epithelial cells, indicating that TGF-β contributed importantly to the initial inflammatory response in the genital mucosa (68).

The effect of TGF-β on myeloid cell differentiation has been associated with the development of LCs, since TGF-β is required for the development of LCs *in vivo* and *in vitro* from CD34+ as well as CD1c+ blood precursors (69–71). In inflammatory conditions, LCs could be derived from circulating monocytes (72), in a process where TGF-β plays a critical role (73). Thus, our study could be understood as an investigation on the potential disruption elicited by PGE2 on the differentiation of LCs. A seminal paper by Geissmann et al. reported that addition of TGF-β to monocytes incubated with IL-4 and GM-CSF leads to a LC-like phenotype characterized by high expression of CD1a and E-cadherin (74). Consistent with our results, these cells were characterized by their inability to express IL-10 when stimulated by LPS, CD40-ligand, or a cytokine cocktail including TNF-α and IL-1β (17). As a matter of fact, their inability to produce IL-10 upon stimulation, while retaining their ability to produce IL-12, has been the most consistent characteristic observed in TGF-β-derived DCs (17, 18, 75, 76). By contrast, their maturation phenotype and ability to induce T cell proliferation are less consistent and appear to vary depending on TGF-β concentration and the stimuli used for activation (17, 47, 76, 77). Most studies used concentrations higher than 2 ng/ml of TGF-β and found a reduced expression of CD80, CD86, and CD83, as well as an attenuated induction of T cell proliferation (17, 47, 76). In our hands, monocytes differentiated in the presence of 0.25 ng/ml of TGF-β still retained the ability to upregulate coestimulatory molecules after LPS stimulation, whereas differentiation at 2.5 ng/ml abrogated this capacity, in agreement with a previous study that also observed concentration-dependent effects of TGF-β on the phenotype of DCs (77). We also observed contrasting effects of high and low dose of TGF-β on IL-12 and IL-23 production, further emphasizing the point that TGF-β acts on monocytes in a concentration-dependent mode. When analyzing DCs differentiated with PGE2, we also noted differences between treatment with lower and higher concentrations of TGF-β. The antagonizing effect of PGE2 was less marked against higher TGF-β levels (2.5 ng/ml) particularly on the proportion of CD1a− CD14+ cells, basal and LPS-induced expression of CD86, and the cytokine expression pattern. These results are consistent with a competition between PGE2 and TGF-β signaling pathways, in which higher levels of TGF-β are needed to counteract increasing levels of PGE2.

Nonetheless, the antagonism exerted by PGE2 might be physiologically relevant in view of the available data regarding TGF-β levels *in vivo*. The actual concentration of active TGF-β at inflammation or tumor sites is still debated, given that its activation is a regulated process and the extracellular matrix acts as a reservoir of latent TGF-β, enabling local spikes of TGF-β levels not reflected by measurements in plasma (78, 79). Total TGF-β levels in inflammatory fluids fluctuate, for instance, between 1.48 ng/ml in ascites from hepatocarcinoma cirrhotic patients, 2 ng/ml in ascites from ovarian cancer patients, and 9.75 ng/ml in pleural effusion of pneumonia patients (42, 43, 45). However, activated TGF-β levels are substantially lower. In the hepatocarcinoma cirrhotic patients, active TGF-β1 in ascites averaged 0.001 ng/ml (42). In other study analyzing human synovial fluid, Albro et al. found an average of 2 ng/ml of total TGF-β but negligible amounts of the active form, which rose to 0.3 ng/ml after 2 h of mechanical activation (44).

When studied in non-immune experimental systems, PGE2 and TGF-β commonly displayed antagonism, as illustrated by PGE2 inhibition of TGF-β-induced collagen synthesis in lung fibroblasts, epithelial–mesenchymal transition in breast cancer or TGF-β-induced differentiation of myofibroblasts (26–28, 80). However, PGE2 has shown synergistic effects with TGF-β on a number of cases, most notably the induction of FoxP3 expression in CD4+ T lymphocytes [(32, 33) and unpublished observations]. The difference between the synergy in FoxP3 expression and the antagonism described in this paper could be explained by a model put forward by Schiller et al. when they studied the interaction of cAMP with the TGF-β transduction pathway in fibroblasts (38). Canonical signaling by TGF-β induces Smad2/3 phosphorylation and entry into the nucleus, modulating downstream gene expression (9). On the other hand, PGE2 receptors EP2 and EP4 are coupled to activation of cAMP-induced PKA and also of PI3K, both of which are capable of phosphorylating CREB transcription factor (81). Schiller et al. proposed that, for some genes, cAMP-induced CREB competed with TGF-β-induced Smad3 for the same transcriptional coactivator (CBP/p300), whereas for other set of genes, in which Smad binding sites and CRE sites coexist, there is a synergistic effect. In agreement with this model, the FoxP3 locus presents Smad binding sites as well as CREB/ATF binding sites in the enhancer region located in intron 2 (82–84). When studying the antagonism in monocytes, in accordance with the competition model, we found TGF-β-induced accumulation of Smad2/3 in the nucleus regardless of the presence of PGE2. In addition, we demonstrated that PGE2 interference of TGF-β could be mimicked by cAMP-elevating agents and could be blocked by inhibition of PKA activity. Of note, we did observe a concentration-dependent effect when we used different amounts of forskolin to compete TGF-β signaling, something that Schiller et al. (38) did not investigate. In this sense, a second study on fibroblast using physiological (lower) levels of cAMP was not able to antagonize TGF-β-induced gene expression (85), further emphasizing the idea that the outcome is concentration-dependent.

A mechanism based on competition that depends on a variable ratio of concentrations of PGE2 and TGF-β could explain, at least partially, the heterogeneity observed in inflammatory DCs. For instance, when characterizing inflammatory DCs from synovial fluid and tumor ascites, Segura et al. have shown the existence of two subpopulations represented by CD16+ CD1c− cells and CD16− CD1c+ cells (5). These subpopulations overlap with our results where TGF-β promotes the CD16− CD1c+ phenotype while PGE2 promotes the other one. Notably, this phenotypic similarity extends to expression of markers HLA-DR, CD11b, and CD206 in both populations. In a different scenario, tumor DCs are also known as a heterogeneous population, where the presence of immunosuppressive tumor-promoting DCs associated with PGE2 is firmly established, although it is also clear that they coexist with several highly immunogenic types (86, 87). Particularly, expression of CD1a has been used to classify tumor DCs, for example, in breast and colorectal cancer (88). Interestingly, it has also been shown that tumor-derived prostanoids inhibit CD1a expression in DCs, even in the presence of TGF-β, further emphasizing the idea that PGE2 plays a defining role in the phenotype of DCs (62, 89, 90).

In summary, our results strongly suggest that the role of TGF-β in the differentiation of DCs is curtailed by the presence of PGE2, which might be often the case in physiological and pathological conditions. While our work concentrated on DCs, it remains to be seen whether PGE2 also interferes TGF-β during the differentiation of other monocyte-derived myeloid cells, such as inflammatory and some tissue-resident macrophages. All in all, the data presented in this article underscores the complexity of myeloid cell differentiation, a dynamic process in which changing concentrations of various factors add up to define the final outcome. In this context, the heterogeneity usually observed in cell populations from biological samples could be understood as a reflection of the history of factors sensed by each individual cell. Further work addressing this complexity is needed to eventually manipulate DCs for their use in cancer, autoimmune, and transplant therapies.

## Ethics Statement

This study is exempt from IRB evaluation, in accordance with the funding agency ethical requirements and also according to current NIH policy on human subject protection, given that the following conditions were met: all the human blood samples used in this research would have been obtained even if the current study was not carried out. Blood samples have not been collected specifically for this research. Blood samples were supplied without personal identifiable information and none of the authors have any ready means to link the materials back to the subjects. In addition, following procedure of the Blood Bank, informed consent for use of samples in research was obtained from all donors whose blood was used in this study.

## Author Contributions

FL designed and performed the experiments, analyzed data, and wrote the manuscript; AP, MP, AV, AM, and CP performed some experiments and analyzed data; JS and JG contributed to experimental design, data analysis and revised the manuscript; and AC designed the experiments, analyzed data, and wrote the manuscript.

## Conflict of Interest Statement

The authors declare that the research was conducted in the absence of any commercial or financial relationships that could be construed as a potential conflict of interest.

## Abbreviations

cAMP, cyclic adenosine mono-phosphate; CFSE, carboxyfluorescein succinimidyl ester; COX2, prostaglandin-endoperoxide synthase-2; CREB, cAMP-responsive-element binding protein; DC, dendritic cell; MDSC, myeloid-derived suppressor cell; PGE2, prostaglandin E2; PKA, protein kinase A; PI3K, phosphatidylinositol-3-phosphate kinase; TGF-β, transforming growth factor-beta.
