# Supplementary material for: Prostaglandin E2 Antagonizes TGF-β Actions During the Differentiation of Monocytes Into Dendritic Cells
Source: Front Immunol. 2018 Jun 22;9:1441. doi: 10.3389/fimmu.2018.01441 (PMC6023975; doi:10.3389/fimmu.2018.01441)
Supplement: Supplementary file 1 [file image_1.PDF]

Supplementary figure 1.

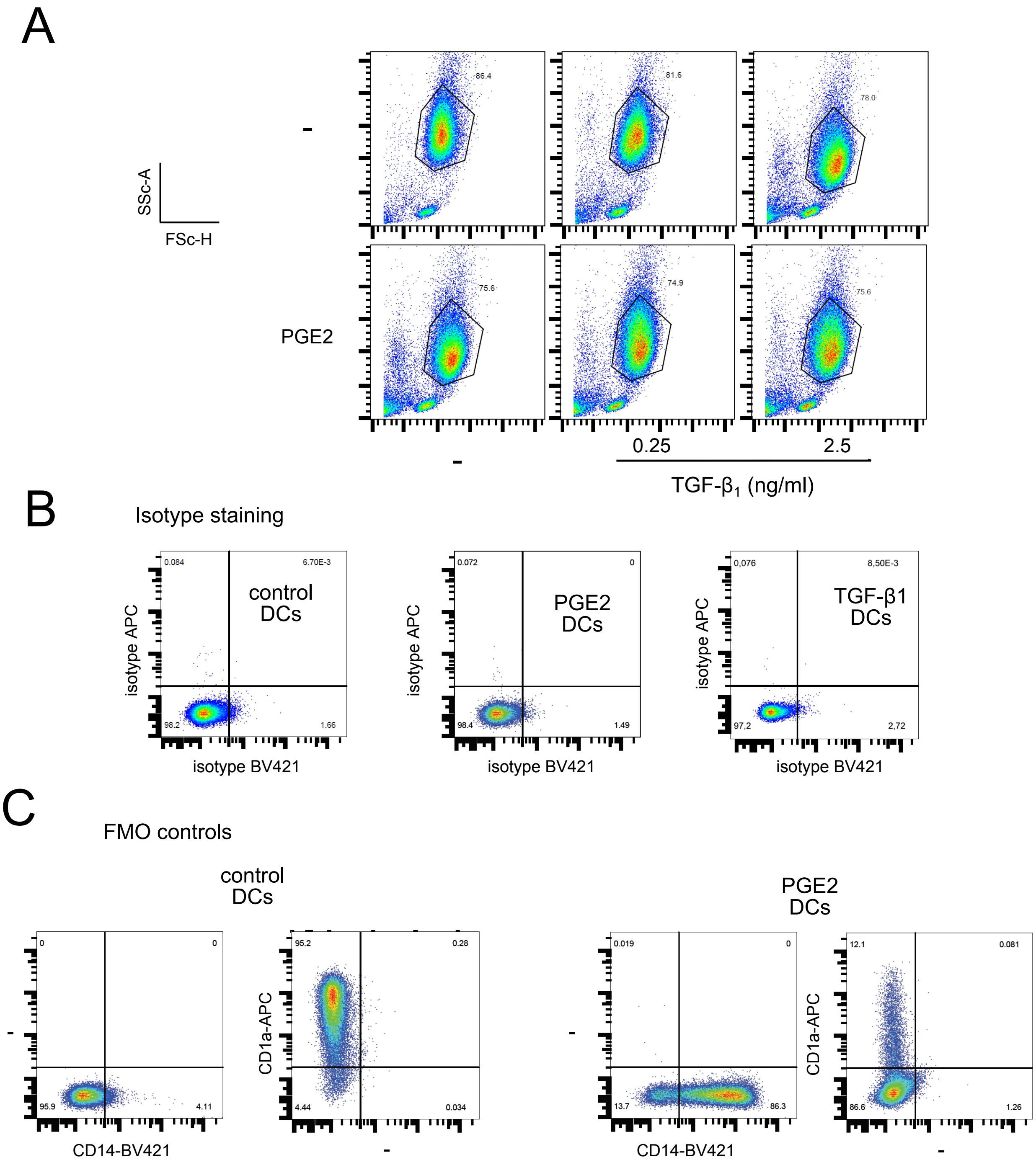

Supplementary Figure 1. Pre-gating, isotype staining and FMO controls for CD1a and CD14 expression.

Monocytes were incubated for 5 days with IL-4 and GM-CSF (control DCs), in the presence of TGF-β (0.25 and 2.5 ng/ml), PGE2 (10^{-7}M), or a combination of both. At day 5, cells were collected for flow cytometry. **(A)** Representative dot plots showing FSc vs SSc. **(B)** Representative dot plots showing isotype staining (IgG1-BV421 and IgG1-APC) for control DCs and DCs differentiated in the presence of PGE2 or TGF-β. **(C)** Representative dot plots showing fluorescence minus one (FMO) staining (minus CD14-BV421 and minus CD1a-APC) for control DCs and DCs differentiated in the presence of PGE2.
