# Supplementary material for: Prostaglandin E2 Antagonizes TGF-β Actions During the Differentiation of Monocytes Into Dendritic Cells
Source: Front Immunol. 2018 Jun 22;9:1441. doi: 10.3389/fimmu.2018.01441 (PMC6023975; doi:10.3389/fimmu.2018.01441)
Supplement: Supplementary file 2 [file image_2.PDF]

Supplementary figure 2.

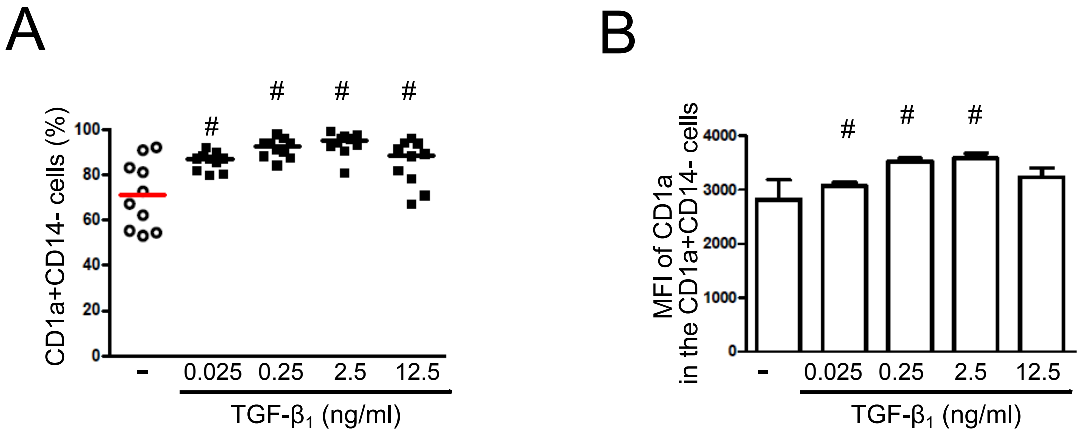

Supplementary Figure 2. Modulation of CD1a expression by TGF-β.

Monocytes were incubated for 5 days with IL-4 and GM-CSF, in the presence or not of TGF-β (concentrations as indicated). At day 5, expression of CD1a and CD14 was analysed by flow cytometry. **(A)** Results from individual donors are expressed as % of CD1a+CD14- cells with median bar (n = 10). **(B)** Quantification of CD1a expression in the CD1a+CD14- subpopulation, showed as MFI (mean ± SEM, n = 10). # indicates $p<0.05$ as calculated after repeated-measures one-way ANOVA analysis.
