# Supplementary material for: Prostaglandin E2 Antagonizes TGF-β Actions During the Differentiation of Monocytes Into Dendritic Cells
Source: Front Immunol. 2018 Jun 22;9:1441. doi: 10.3389/fimmu.2018.01441 (PMC6023975; doi:10.3389/fimmu.2018.01441)
Supplement: Supplementary file 3 [file image_3.PDF]

Supplementary figure 3.

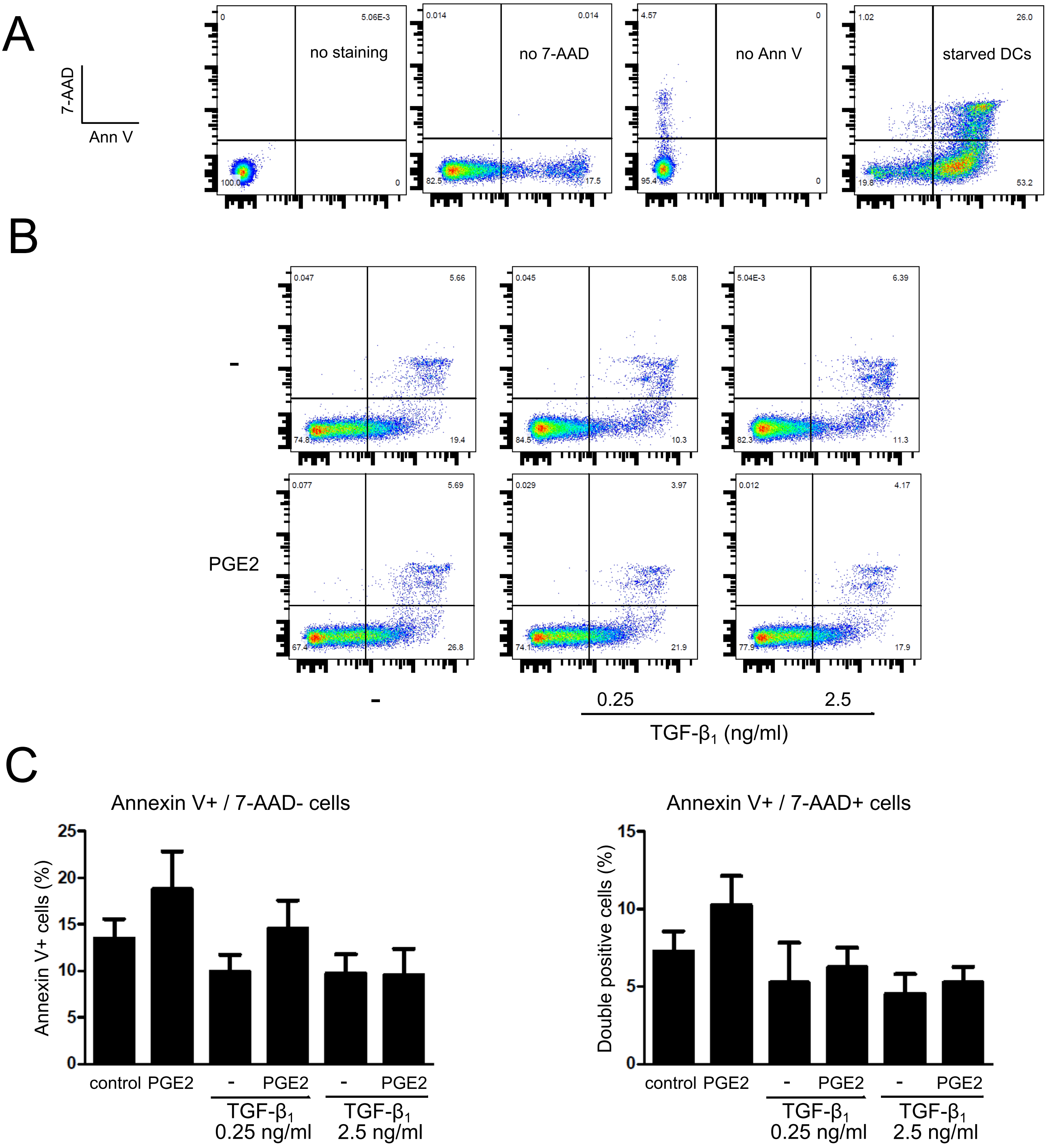

Supplementary Figure 3. DC differentiation in the presence of TGF-β, PGE2 or both, does not affect viability of DCs.

Monocytes were incubated for 5 days with IL-4 and GM-CSF (control DCs), in the presence of TGF-β (0.25 and 2.5 ng/ml), PGE2 (10^{-7}M), or a combination of both. At day 5, viability was evaluated by annexin V and 7-AAD staining followed by flow cytometry **(A)** Dot blots showing staining controls, including serum-starved (12 hours) DCs. **(B)** Representative dot plots showing annexin V and 7-AAD staining.. **(C)** Quantification of viability as % of annexin V+ cells or as double positive annexin V/7-AAD cells (mean ± SEM, n=3).
