# Supplementary material for: Prostaglandin E2 Antagonizes TGF-β Actions During the Differentiation of Monocytes Into Dendritic Cells
Source: Front Immunol. 2018 Jun 22;9:1441. doi: 10.3389/fimmu.2018.01441 (PMC6023975; doi:10.3389/fimmu.2018.01441)
Supplement: Supplementary file 4 [file image_4.PDF]

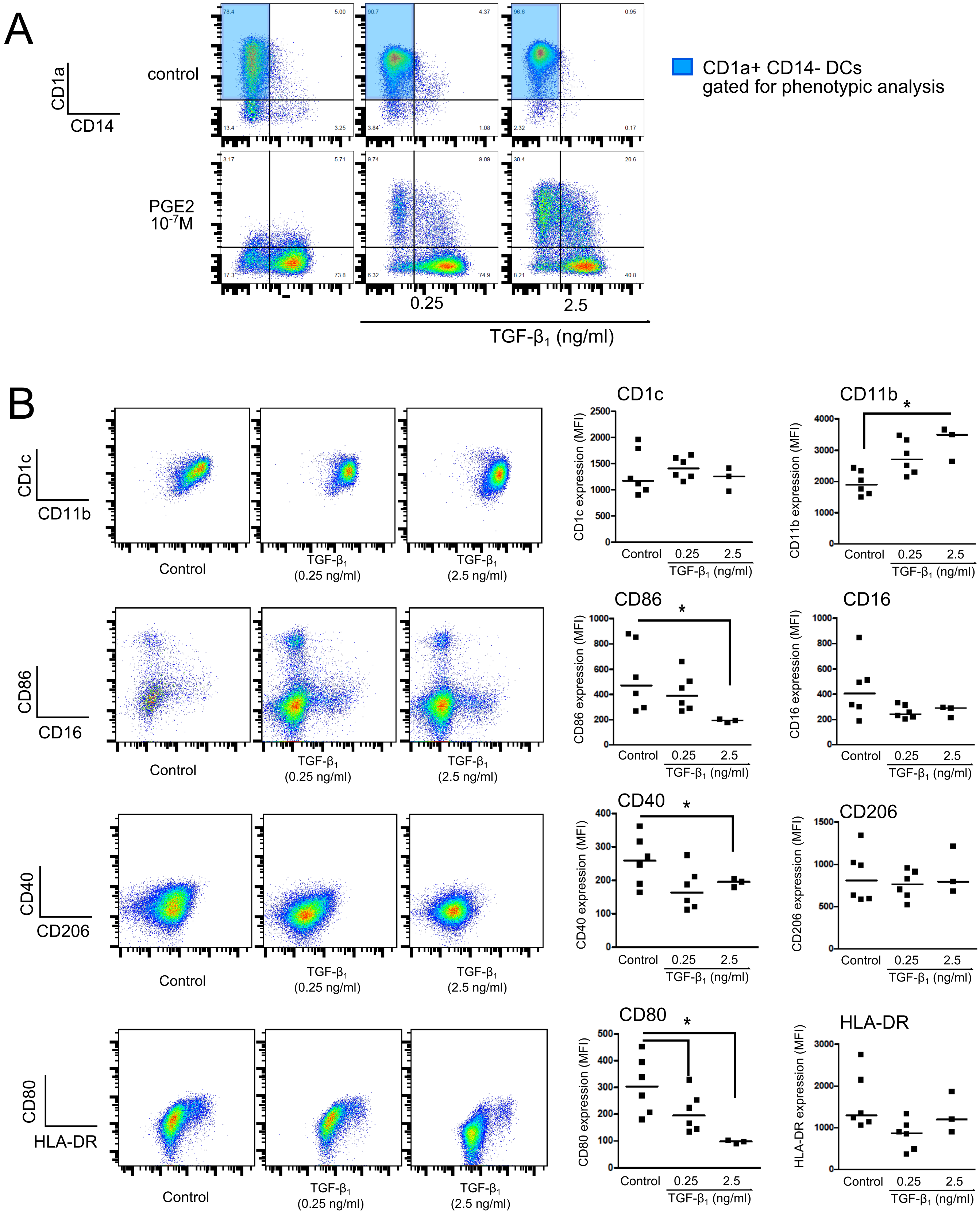

Supplementary Figure 4. Phenotypic signature of control DCs and DCs differentiated in the presence of TGF-β. Monocytes were incubated for 5 days with IL-4 and GM-CSF (control DCs) or in the presence of TGF-β (0.25 and 2.5 ng/ml). **(A)** Representative dot plots for expression of CD1a and CD14, showing gating of the CD1a+CD14- subpopulation for further phenotypic analysis. **(B)** Representative dot plots and quantification showing expression of selected markers, analyzed in the CD1a+CD14- subpopulation. Results from individual donors are expressed as MFI with median bar (n = 3-6). * indicates $p<0.05$ as calculated after one-way ANOVA test, followed by Bonferroni posttest for selected comparisons. MFI indicates Mean Fluorescence Intensity.
