# Supplementary material for: Prostaglandin E2 Antagonizes TGF-β Actions During the Differentiation of Monocytes Into Dendritic Cells
Source: Front Immunol. 2018 Jun 22;9:1441. doi: 10.3389/fimmu.2018.01441 (PMC6023975; doi:10.3389/fimmu.2018.01441)
Supplement: Supplementary file 5 [file image_5.PDF]

Supplementary figure 5.

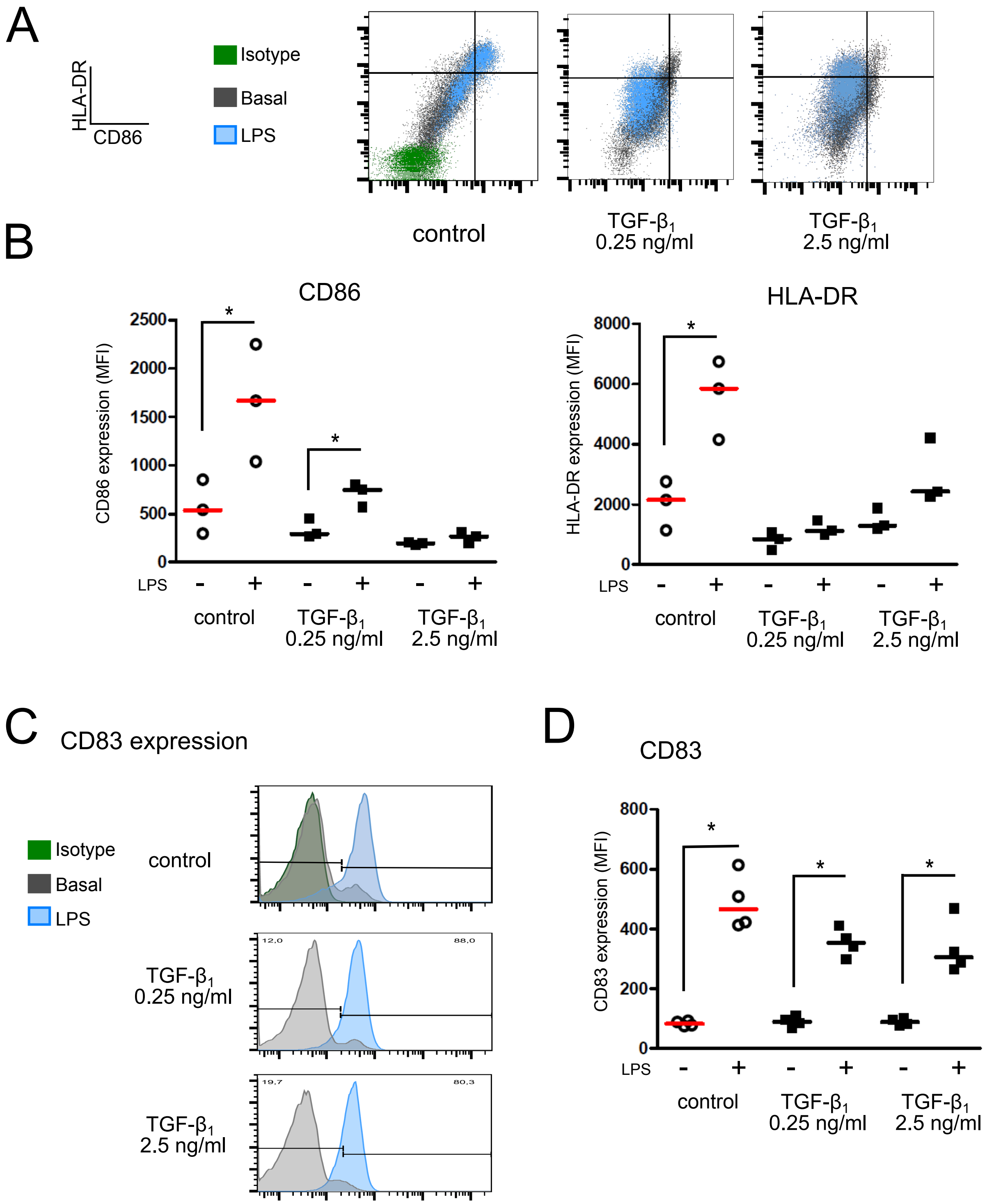

Supplementary Figure 5. Presence of TGF-β during differentiation of DCs impairs LPS-induced increase of CD86 and HLA-DR, but does not affect CD83.

Monocytes were incubated for 5 days with IL-4 and GM-CSF with or without addition of TGF-β (0.25 and 2.5 ng/ml). Further analysis was performed in the gated CD1a+CD14- subpopulation. Next, DCs were washed and exposed for 24 h to LPS (20 ng/ml). **(A)** Representative dot plots for expression of CD86 and HLA-DR, analyzed in the CD1a+CD14- subpopulation. **(B)** Results from individual donors are expressed as MFI with median bar (n = 3). **(C)** Representative histograms for expression of CD83, analyzed in the CD1a+CD14- subpopulation. **(D)** Results from individual donors are expressed as MFI with median bar (n = 4). In all cases, * indicates $p<0.05$ as calculated after repeated-measures one-way ANOVA analysis. MFI indicates Mean Fluorescence Intensity.
