# Supplementary material for: Prostaglandin E2 Antagonizes TGF-β Actions During the Differentiation of Monocytes Into Dendritic Cells
Source: Front Immunol. 2018 Jun 22;9:1441. doi: 10.3389/fimmu.2018.01441 (PMC6023975; doi:10.3389/fimmu.2018.01441)
Supplement: Supplementary file 6 [file image_6.PDF]

Supplementary figure 6.

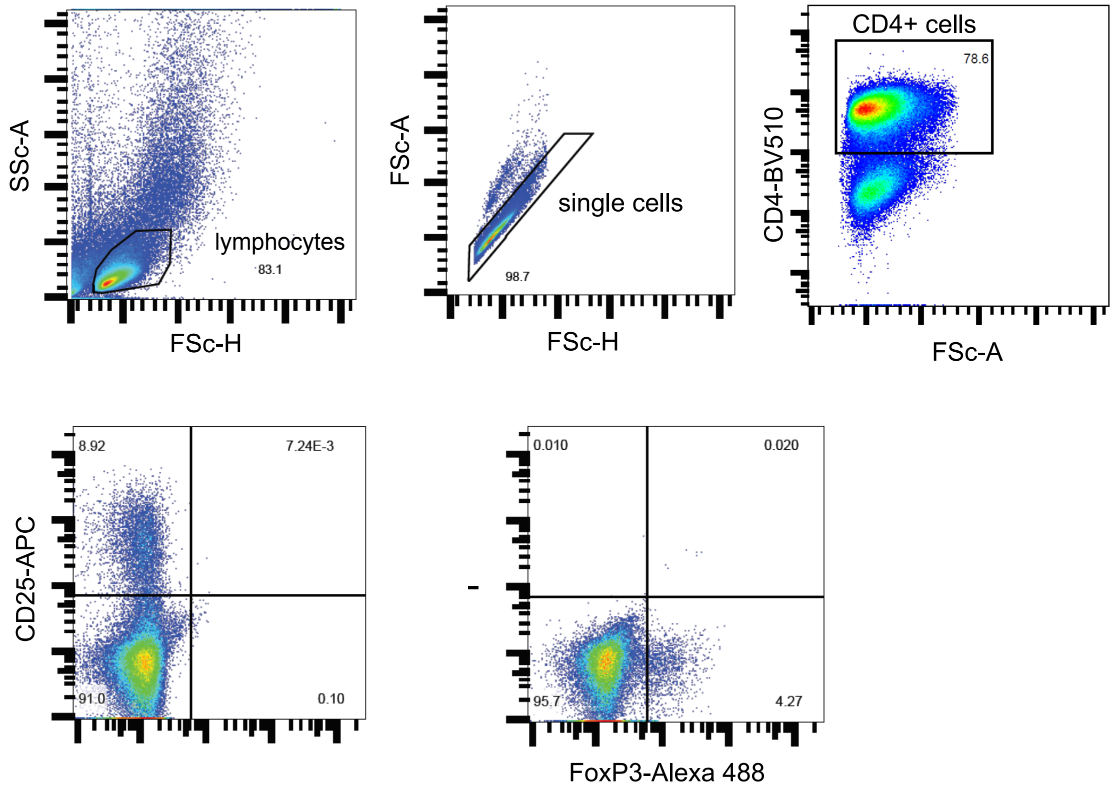

Supplementary Figure 6. Pre-gating and FMO controls for CD25 and FoxP3 expression.

CD4+ T cells were purified and incubated with LPS-stimulated control DCs. At day 4, cells were collected for flow cytometry. **(A)** Representative dot plots of FSc vs SSc, single cell gating (FSc-H vs FSc-A) and CD4+ gating. **(B)** Representative dot plots showing fluorescence minus one (FMO) staining controls (minus anti-FoxP3-Alexa 488 and minus anti-CD25-APC) for lymphocytes co-cultured with control DCs.
